# Early T Cell Recognition of B Cells following Epstein-Barr Virus Infection: Identifying Potential Targets for Prophylactic Vaccination

**DOI:** 10.1371/journal.ppat.1005549

**Published:** 2016-04-20

**Authors:** Jill M. Brooks, Heather M. Long, Rose J. Tierney, Claire Shannon-Lowe, Alison M. Leese, Martin Fitzpatrick, Graham S. Taylor, Alan B. Rickinson

**Affiliations:** 1 Institute of Cancer and Genomic Sciences, University of Birmingham, Birmingham, United Kingdom; 2 Institute of Immunology and Immunotherapy, University of Birmingham, Birmingham, United Kingdom; 3 Biomolecular Mass Spectrometry and Proteomics Group, Utrecht University, Utrecht, The Netherlands; Baylor College of Medicine, UNITED STATES

## Abstract

Epstein-Barr virus, a B-lymphotropic herpesvirus, is the cause of infectious mononucleosis, has strong aetiologic links with several malignancies and has been implicated in certain autoimmune diseases. Efforts to develop a prophylactic vaccine to prevent or reduce EBV-associated disease have, to date, focused on the induction of neutralising antibody responses. However, such vaccines might be further improved by inducing T cell responses capable of recognising and killing recently-infected B cells. In that context, EBNA2, EBNA-LP and BHRF1 are the first viral antigens expressed during the initial stage of B cell growth transformation, yet have been poorly characterised as CD8+ T cell targets. Here we describe CD8+ T cell responses against each of these three “first wave” proteins, identifying target epitopes and HLA restricting alleles. While EBNA-LP and BHRF1 each contained one strong CD8 epitope, epitopes within EBNA2 induced immunodominant responses through several less common HLA class I alleles (e.g. B*3801 and B*5501), as well as subdominant responses through common class I alleles (e.g. B7 and C*0304). Importantly, such EBNA2-specific CD8+ T cells recognised B cells within the first day post-infection, prior to CD8+ T cells against well-characterised latent target antigens such as EBNA3B or LMP2, and effectively inhibited outgrowth of EBV-transformed B cell lines. We infer that “first wave” antigens of the growth-transforming infection, especially EBNA2, constitute potential CD8+ T cell immunogens for inclusion in prophylactic EBV vaccine design.

## Author Summary

Epstein-Barr virus infects the vast majority of the world’s population; in most individuals both primary infection and long-term virus carriage are asymptomatic. However, EBV is the major cause of glandular fever, is associated with multiple cancers and is implicated in various autoimmune conditions; thus there is a strong impetus for the development of a prophylactic vaccine. To date, vaccine design has largely focused on the induction of neutralising antibodies to virion structural components which can prevent virus binding and infection. Such strategies may be improved by the inclusion of immunogens to induce T cell responses with the potential to promptly recognise and eliminate cells that do become infected. Here we characterise T cell responses to three proteins, EBNA2, EBNA-LP and BHRF1 that comprise the “first wave” of de novo viral antigen expression following EBV infection of B cells. Each of these proteins is targeted by strong T cell responses in a subset of donors. Furthermore, CD8+ T cells specific for that at least one of these proteins, EBNA2, efficiently recognise B cells at very early time-points post-infection, before CD8+ T cells of all other specificities tested, and effectively inhibit outgrowth of B cell lines following EBV infection in vitro. Thus “first wave antigens”, particularly EBNA2, may comprise suitable candidate immunogens for inclusion in prophylactic EBV vaccine design.

## Introduction

Epstein-Barr virus (EBV), a human γ-herpesvirus with potent B cell growth-transforming ability, is carried by most individuals as an asymptomatic infection yet has a remarkable potential to cause disease. Thus delayed primary infection of the immune-competent host leads in many cases to infectious mononucleosis (IM), where disease symptoms are coincident with an over-active T cell response [1]; while infection of T cell-compromised or T cell-suppressed patients brings a high risk of EBV-driven B-lymphoproliferative disease (LPD). Equally important, the virus is linked to a number of lymphoid and epithelial malignancies that arise as a consequence of longer-term virus carriage [2,3]. Collectively these EBV genome-positive tumours, including endemic Burkitt Lymphoma, many cases of Hodgkin Lymphoma, adult T/NK cell lymphoma, nasopharyngeal carcinoma and a subset of gastric carcinomas, impose a global disease burden of ~200,000 new cancer cases per year [4]. EBV infection is also implicated as a major environmental risk factor for the development of various autoimmune conditions, especially Multiple Sclerosis [5].

Viewed in this light, there is a compelling case for a prophylactic vaccine that could either prevent EBV infection altogether or, at least, reduce disease risk by lowering the set-point of long-term virus carriage [6,7]. However there is uncertainty about which viral antigens to include in such a vaccine, not least because the very early events of in vivo infection are poorly understood. The virus is orally transmitted and replicates in the oropharynx, probably in mucosal epithelium and/or locally-infiltrating B cells, while colonising the lymphoid system via the expansion of virus-transformed cells before entering a true (antigen-negative) latency in the recirculating memory B cell pool [8]. Arguably all EBV-associated diseases depend directly or indirectly on this initial colonisation of the B cell system [9]; thus all available evidence suggests that the virus does not persist at epithelial sites and that all non-B cell-derived tumours arise from infections acquired by reactivation from the latent B cell reservoir. The aim of a prophylactic vaccine must therefore be to prevent or limit the virus’ initial colonisation of the B cell system.

Most interest in that regard has focused on the initial process of B cell infection, where viral attachment is mediated by the major envelope glycoprotein gp350 binding to the complement receptor CD21 on the B cell surface. Thereafter viral entry involves binding of a co-receptor (HLA class II) by the gp85/gp25/gp42 glycoprotein complex, and a subsequent envelope fusion event requiring gp85/gp25 and another viral glycoprotein gp110 [10]. Vaccine constructs based on gp350, known to be the dominant target of the neutralising antibody response, have been tested in primate models [11] and more recently in man [12,13]. In the only phase II clinical trial, a recombinant gp350 vaccine given to EBV-naïve adolescent volunteers apparently did not lower the chances of becoming infected but did reduce the number of primary infections manifesting as IM [13]. However, the mechanism underlying disease protection (neutralising antibodies and/or T cell-mediated immunity) and the impact of vaccination on the long-term viral load in the B cell system were not addressed. A small trial in monkeys studying the EBV homologue, rhesus lymphocryptovirus, found that gp350 vaccination reduced both incidence of infection and long-term viral load after oral challenge. Furthermore, the inclusion of EBNA3A/3B in vaccine constructs induced T cell responses which appeared to mediate additional protection [14]. EBNA3A was also targeted in the sole phase I trial of a CD8+ T cell peptide epitope-based vaccine for IM [15]. We inferred from the work to date that a gp350-based EBV vaccine, even optimised to induce higher neutralising antibody responses [16] might be further improved by eliciting T cell responses, in particular CD8+T cell responses, capable of recognising and killing recently-infected B cells in the very early stages of virus-induced transformation.

There are three possible sources of such T cell target antigens:

1. virus structural proteins that are expressed late in the lytic cycle, incorporated into progeny virions and delivered as virion components into the resting B cell. In this context previous studies have shown that natural virus infection elicits CD4+ T cell responses to many late structural proteins [17–19], and that de novo-infected B cells can present such proteins via the HLA class II pathway for recognition by late antigen-specific CD4+ T cell clones [17]. By contrast these same proteins naturally elicit weak CD8+ T cell responses [20,21], and whether recently-infected B cells are capable of cross-presenting these exogenously acquired structural proteins to CD8+ T cells remains in doubt.
2. other lytic cycle proteins, whose reported expression in the first 1–2 days post-infection [22–25] has recently been attributed to the translation of viral mRNAs carried into the resting B cell within incoming virions [26]. Such proteins include immediate early (IE) and early (E) lytic antigens (BZLF1/BRLF1 and BMLF1/BMRF1 respectively) that naturally constitute strong CD8+ T cell targets [20,27].
3. latent proteins of the growth-transforming programme itself. These are expressed in three successive waves from the incoming virus genome [28]. Firstly, activation of the Wp promoter drives expression of two transcriptional activators, the nuclear antigens EBNA2 and EBNA-LP, and the viral bcl2 homologue BHRF1. Thereafter transcription switches to the alternative pan-EBNA promoter Cp, broadening EBNA expression to include EBNAs 1, 3A, 3B and 3C. Subsequently activation of a bi-directional promoter drives expression of the latent membrane proteins LMP1 and LMP2, whose appearance 3–4 days post-infection coincides with blast transformation, the onset of cell division and, particularly relevant to the present work, the reported LMP1-driven up-regulation of antigen processing pathways [29,30]. Most work to date on CD8+ T cell responses to latent proteins has focused on the immunodominant EBNA3A, 3B, 3C family, and on the subdominant EBNA1 and LMP2 targets [31]. Surprisingly little attention has been given to the EBNA2, EBNA-LP and BHRF1 proteins despite the fact that these constitute the “first wave” of target antigens appearing during B cell transformation.

In the present work, we set out to detect CD8+ T cell responses against these “first wave” transforming proteins, to identify their target epitopes and HLA restricting alleles, and to determine how well they recognised B cells in the days following EBV infection, comparing such recognition with that shown by CD8+ T cells against the other two sources of candidate vaccine antigens.

## Results

### Identification of EBNA2, EBNA-LP and BHRF1-specific T cell responses

In a first series of experiments we screened a panel of 20 healthy EBV-seropositive donors for T cell responses to EBNA2, EBNA-LP and BHRF1. Peripheral blood mononuclear cells (PBMCs) were stimulated with peptide pools comprised of overlapping peptides spanning the complete unique amino acid sequence of each of these proteins. Following 7 days of culture in cytokine supplemented medium, the resultant polyclonal T cell populations were screened for recognition of peptide sub-pools; recognition was assessed by IFNγ production measured in Enzyme-linked Immunosorbent assays (ELISAs). In these initial screening experiments we analysed both CD4-selected and CD4-depleted (i.e. CD8-enriched) T cell populations to determine the overall pattern of responses.

**Fig 1:**
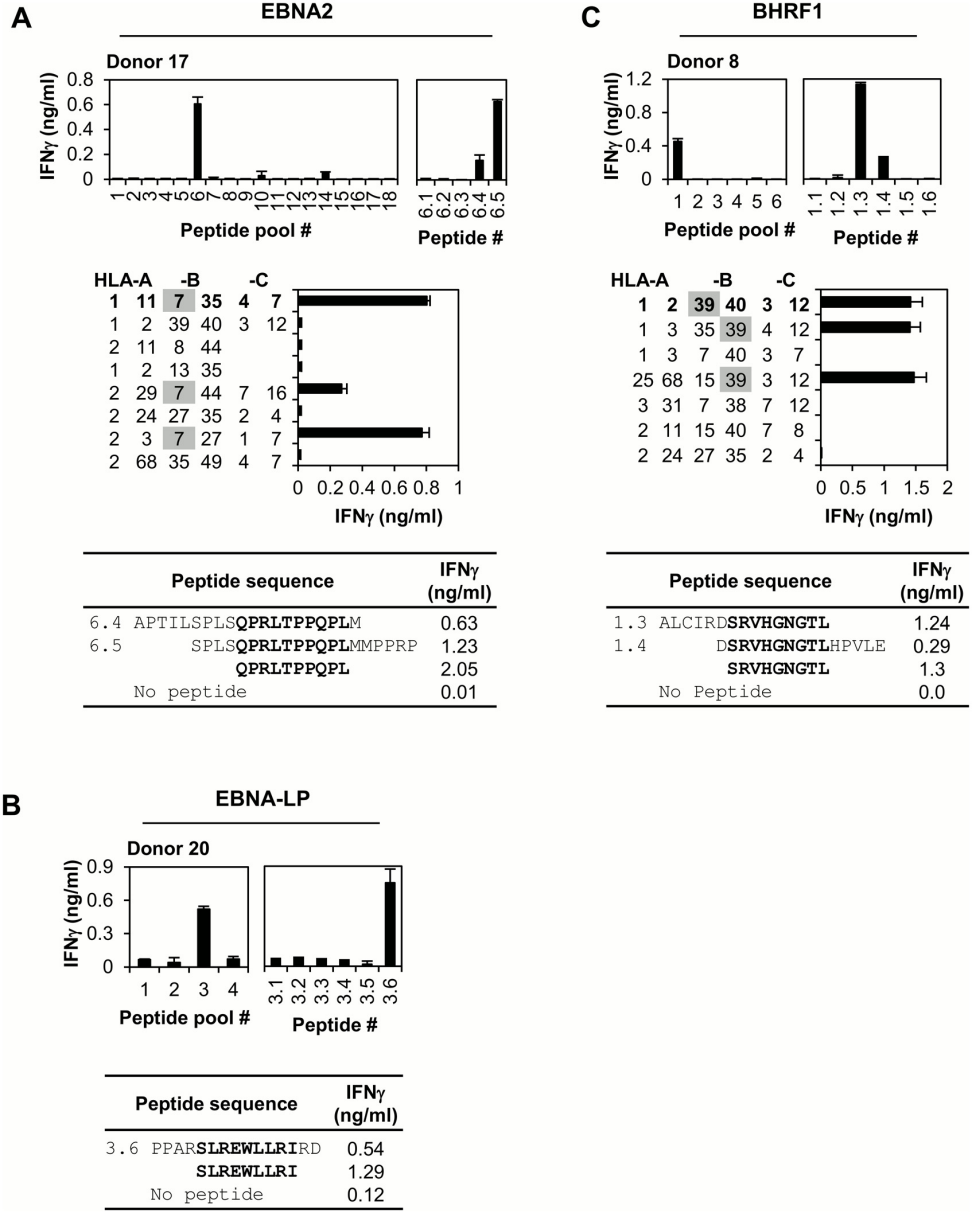
Identification of EBNA2-, EBNA-LP- and BHRF1-specific CD8+ T cell responses. (A) EBNA2-specific responses. Top left panel: In vitro expanded, CD8-enriched polyclonal T cells from Donor 17 were screened against overlapping 20mer peptides spanning the complete unique sequence of EBNA2 (sub-divided into 18 pools). T cell recognition was assessed by IFNγ production, measured in an ELISA. Results are expressed as the mean IFNγ concentration +/- SD for duplicate wells. Top right panel: Individual component peptides from pool 6 were screened for their ability to mediate IFNγ production by the CD8-enriched T cell population. Middle panel: To identify the HLA restriction, LCLs sharing one or more class I alleles with Donor 17 (class I type in bold) were pre-loaded with peptides 6.4/6.5 (1μg/ml) and co-cultured overnight with a specific T cell clone. Results are expressed as the mean IFNγ concentration +/- SD for triplicate wells. Table: Peptides 6.4, 6.5 and the predicted minimal epitope were screened for their ability to induce IFNγ production by the CD8-enriched polyclonal T cell population. (B) EBNA-LP-specific responses. Left panel: In vitro expanded CD8-enriched polyclonal T cells from Donor 20 were screened against overlapping 15mer peptides spanning the complete unique sequence of EBNA-LP (sub-divided into 4 pools); T cell recognition was assessed by IFNγ production. Right panel: Individual component peptides from pool 3 were screened for their ability to induce IFNγ production by the CD8-enriched T cell population. Sequences and T cell recognition data for peptide 3.6 and the minimal epitope are shown in the table. (C) BHRF1-specific responses. Top left panel: In vitro expanded CD8-enriched polyclonal T cells from Donor 8 were screened against overlapping 15mer peptides spanning the complete unique sequence of BHRF1 (sub-divided into 6 pools). Top right panel: Individual component peptides from pool 1 were screened for their ability to mediate IFNγ production by the total polyclonal T cell population. Middle panel: To identify the HLA restriction, LCLs sharing one or more class I alleles with Donor 8 (class I type in bold) were pre-loaded with peptides 1.3/1.4 (1μg/ml) and co-cultured overnight with a specific T cell clone. Results are expressed as the mean IFNγ concentration +/- SD for triplicate wells. Table: Peptides 1.3/1.4 and the predicted minimal epitope were screened for their ability to mediate IFNγ production by the CD8-enriched T cell population.

CD8+ T cell responses to EBNA2: Fig 1A and S1A Fig illustrate the screening strategy for EBNA2-specific responses and the characterisation of novel CD8+ T cell epitopes. In the first example, CD8-enriched T cell populations from Donor 17 were screened against 18 peptide sub-pools representing the unique 487 amino acid sequence of EBNA2 (B95.8 strain); a single response was detected to peptide(s) within pool 6. Subsequent screening against individual peptides from this pool identified two overlapping peptides, 6.4 and 6.5 that mediated T cell recognition (Fig 1A, upper panels). The peptide 6.4/6.5-specific polyclonal population was cloned by limiting dilution and the resultant CD8+ T cell clones used to determine the HLA restriction of this response. Fig 1A (middle panel) illustrates the results obtained for one representative T cell clone screened against HLA class I-matched LCLs, pre-loaded with peptides 6.4/6.5 to maximise recognition; only LCLs sharing HLA-B7 were recognised. The sequence QPRLTPPQPL located within the overlapping region of peptides 6.4 and 6.5 conforms well to the defined HLA-B7 peptide motif. This 10-mer peptide was recognised by both the polyclonal CD8-enriched T cell population (Fig 1A, table) and specific T cell clones. Results from screening a second donor (Donor 8) against the EBNA2 peptide sub-pools are shown in S1A Fig; here the response mapped to the 9mer TSSPSMPEL presented by HLA-C*0304. In total, 19 donors were screened against the EBNA2 peptide sub-pools. CD8+ T cell responses for individual donors are detailed in S1 Table; all EBNA2-derived CD8 responses detected are summarised in Table 1. These include three previously described epitopes, as well as 5 novel CD8+ T cell responses, the HLA class I restriction determinants were identified for three of these responses and two minimal epitopes were defined.

**Table 1:** CD8+ T cell epitopes.

EBV antigen	Epitope	Co-ordinates	HLA restriction
**EBNA2**	YHLIVDTDSL	14–23	B*3801
	**DVGHGPLASAMRMLWMANYI** ^⊥^	121–140	nd
	LASAMRML	127–135	B*5701/B*5801
	**SPLSQPRLTPPQPLMMPPRP** ^⊥^	181–200	B*0702
	**QPRLTPPQPL**	185–194	B*0702
	RPTELQPTP	234–242	B*5501
	**TSSPSMPEL**	377–384	C*0304
	**SMPELSPVLGLHQGQGAGDS** ^⊥^	381–390	nd
**EBNA-LP**	SLREWLLRI	284–292	A*0203
**BHRF1**	**SRVHGNGTL**	17–25	B*3901
	**ETFTETWNR**	63–71	A*6801

Epitopes in bold not previously published.

^⊥^ Minimal epitope not defined.

nd not determined.

CD8+ T cell responses to EBNA-LP: EBNA-LP (B95.8 strain) contains only 110 residues of unique sequence, being comprised of multiple copies of a 66 amino acid repeat domain upstream of a unique 44 amino acid carboxy-terminus. Screening 18 of the above 20 donors, with a range of HLA class I types, against peptide sub-pools covering the whole EBNA-LP sequence gave only a single positive result. The CD8-enriched T cell population from Donor 20 recognised peptide(s) within pool 3, and this response mapped to peptide 3.6, which encompasses the previously identified SLR epitope [32], EBNA-LP amino acids 284–292 (Fig 1B), lying within the unique C-terminal domain. Donor 20 indeed turned out to be HLA-A*0203-positive and the response proved to be HLA-A*0203-restricted.

CD8+ T cell responses to BHRF1: Prior to this work, the CD8+ T cell response to BHRF1 had not been characterised. BHRF1 is another relatively small protein which, in the B95.8 strain of EBV, is comprised of 191 amino acids. Screening 18 donors against 6 BHRF1 peptide sub-pools detected responses to two CD8+ T cell epitopes. As illustrated in Fig 1C, the CD8-enriched T cell population generated from Donor 8 recognised peptide(s) contained within pool 1; this response mapped to peptides 1.3/1.4. HLA restriction analysis identified B*3901 as the restriction determinant; the peptide SRVHGNGTL contained within the 10 amino acid sequence common to peptides 1.3/1.4 conforms well to the B*3901 peptide motif and was identified as the minimal epitope. Results for a different donor mapping the second BHRF1-derived CD8 response to the 9mer ETFTETWNR, presented by HLA-A68, are shown in S1B Fig. T cell clones specific for the two BHRF1-derived epitopes, as well as the two minimally-defined EBNA2 epitopes, recognised their respective proteins expressed from a recombinant vaccinia virus (rVV) confirming their antigen as well as epitope specificity (S1C and S1D Fig). BHRF1-specific CD8+ T cell responses are summarised in Table 1 and detailed for individual donors in S1 Table.

**Fig 2:**
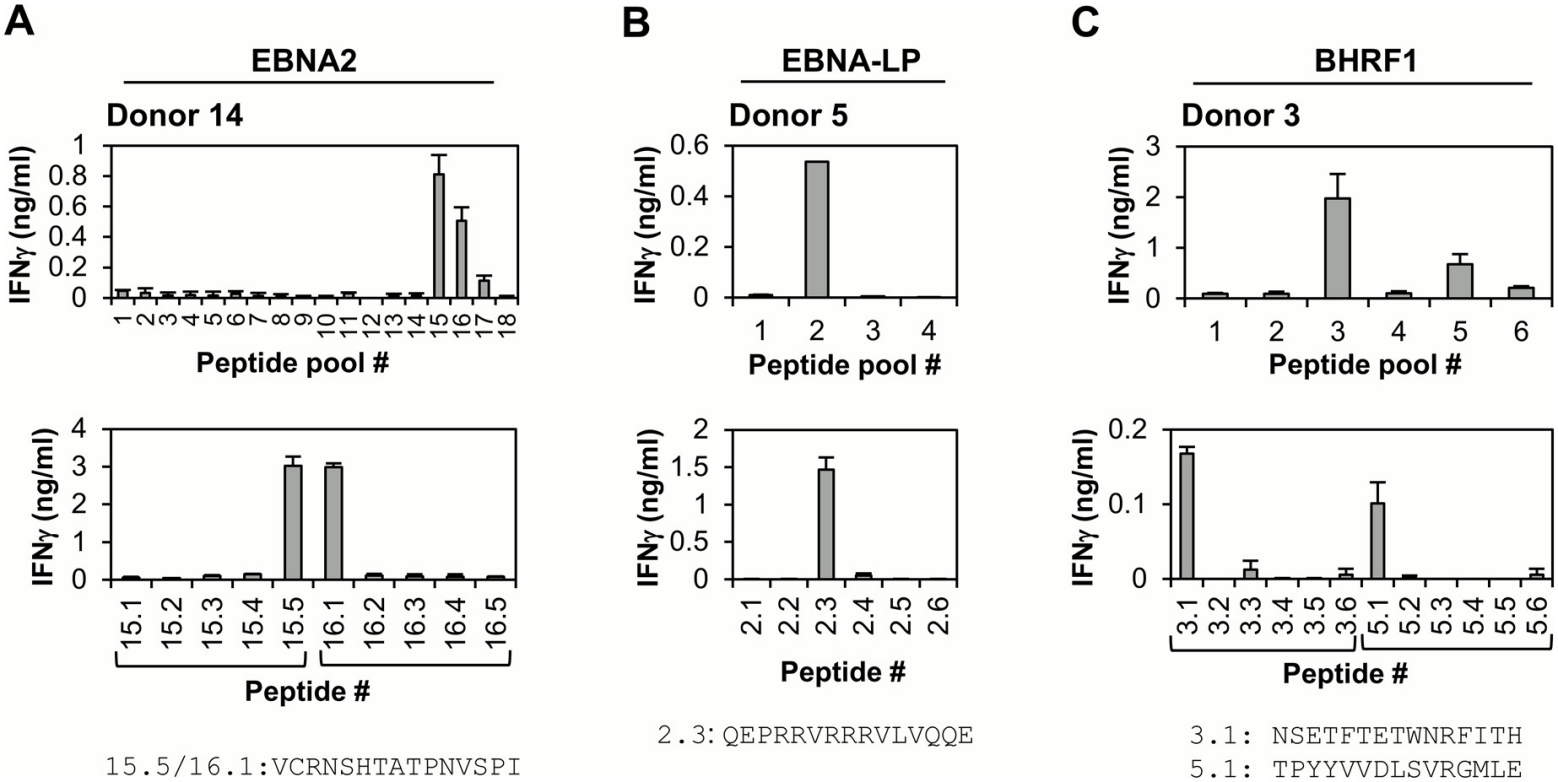
Identification of EBNA2-, EBNA-LP- and BHRF1-specific CD4+ T cell responses. (A) EBNA2-specific responses. Top panel: In vitro expanded CD4-selected polyclonal T cells from Donor 14 were screened for reactivity against overlapping peptides spanning the complete unique sequence of EBNA2. Results are expressed as the mean IFNγ concentration +/- SD for duplicate wells. Lower panel: Individual component peptides from adjacent pools 15 and 16 were screened for their ability to induce IFNγ production by the CD4-selected T cell population. The sequence of the 15mer peptide common to pools 15.5 and 16.1 is given below the figure. (B) EBNA-LP-specific responses. Top panel: In vitro expanded total polyclonal T cells from Donor 5 were screened for recognition of peptide pools spanning the complete unique sequence of EBNA-LP. Bottom panel: Individual component peptides from pool 2 were screened for their ability to mediate IFNγ production by the total T cell population; the sequence of the active peptide, 2.3, is given. Note that this response was abolished following CD4-depletion. (C) BHRF1-specific responses. Top panel: In vitro expanded CD4-selected polyclonal T cells from Donor 3 were screened for reactivity against overlapping peptides spanning BHRF1. Lower panel: Individual peptides from pools 3 and 5 were screened for their ability to induce IFNγ production by the CD4-selected T cell population. The sequence of the two active peptides (3.1 and 5.1) is given.

CD4+ T cell responses: In parallel with the above work, donors were screened for CD4+ T cell responses to these three “first wave” antigens; several new responses were observed. Illustrative results for one donor per protein are shown in Fig 2. Thus for EBNA2, the CD4-selected T cell population from Donor 14 recognised peptides from two adjacent sub-pools, 15 and 16; splitting these pools into their component peptides narrowed the response down to two overlapping peptides 15.5 and 16.1, which share the sequence VCRNSHTATPNVSPI (Fig 2A). Of 19 donors included in the screening, 17 (~ 90%) had detectable EBNA2-specific CD4+ T cell responses (S1 Table), although such responses were not always characterised beyond the peptide pool(s) recognised. The two responses defined at the individual peptide level are included in Table 2, which summarises all EBNA2-derived CD4 responses detected in this study. We found that, despite its small size, EBNA-LP was also a relatively frequent target of CD4+ T cell responses, often directed against a shared immunodominant epitope. Thus 7/18 donors recognised EBNA-LP sub-pool 2 and, of these, all three donors screened further recognised peptide 2.3, sequence QEPRRVRRRVLVQQE. This is illustrated for one representative donor (Donor 5) in Fig 2B. Responsive donors do not share a common class II allele, suggesting that this peptide is presented by multiple class II antigens. Finally, screening 18 donors against the BHRF1 peptide pools identified one novel and one known CD4 epitope response (Table 2). For example, as shown in Fig 2C the CD4-selected T cell population from Donor 3 recognised peptides within pools 3 and 5. Analysis of individual pool 3 peptides mapped the response to peptide 3.1, sequence NSETFTETWNRFITH; the pool 5 response mapped to peptide 5.1, which corresponds to the published PYY epitope [33]; BHRF1-specific CD4 responses for individual donors are summarised in S1 Table.

**Table 2:** CD4+ T cell epitopes.

EBV antigen	Epitope	Co-ordinates	HLA restriction
**EBNA2**	FVGENTGVPPPLPPP	51–65	nd
	**TPLLTVLQRPTELQLSPLSQ**	226–245	**nd**
	PRSPTVFYNIPPMPLPPSQL	276–295	DR7, 52a,52b,52c
	PAQPPPGVINDQQLHHLPSG	301–320	DR17
	**VCRNSHTATPNVSPIHEPES**	411–425	nd
**EBNA-LP**	**QEPRRVRRRVLVQQE**	41–55	multiple
**BHRF1**	**NSETFTETWNRFITH**	61–75	nd
	PYYVVDLSVRGM	122–133	DR4

**Fig 3:**
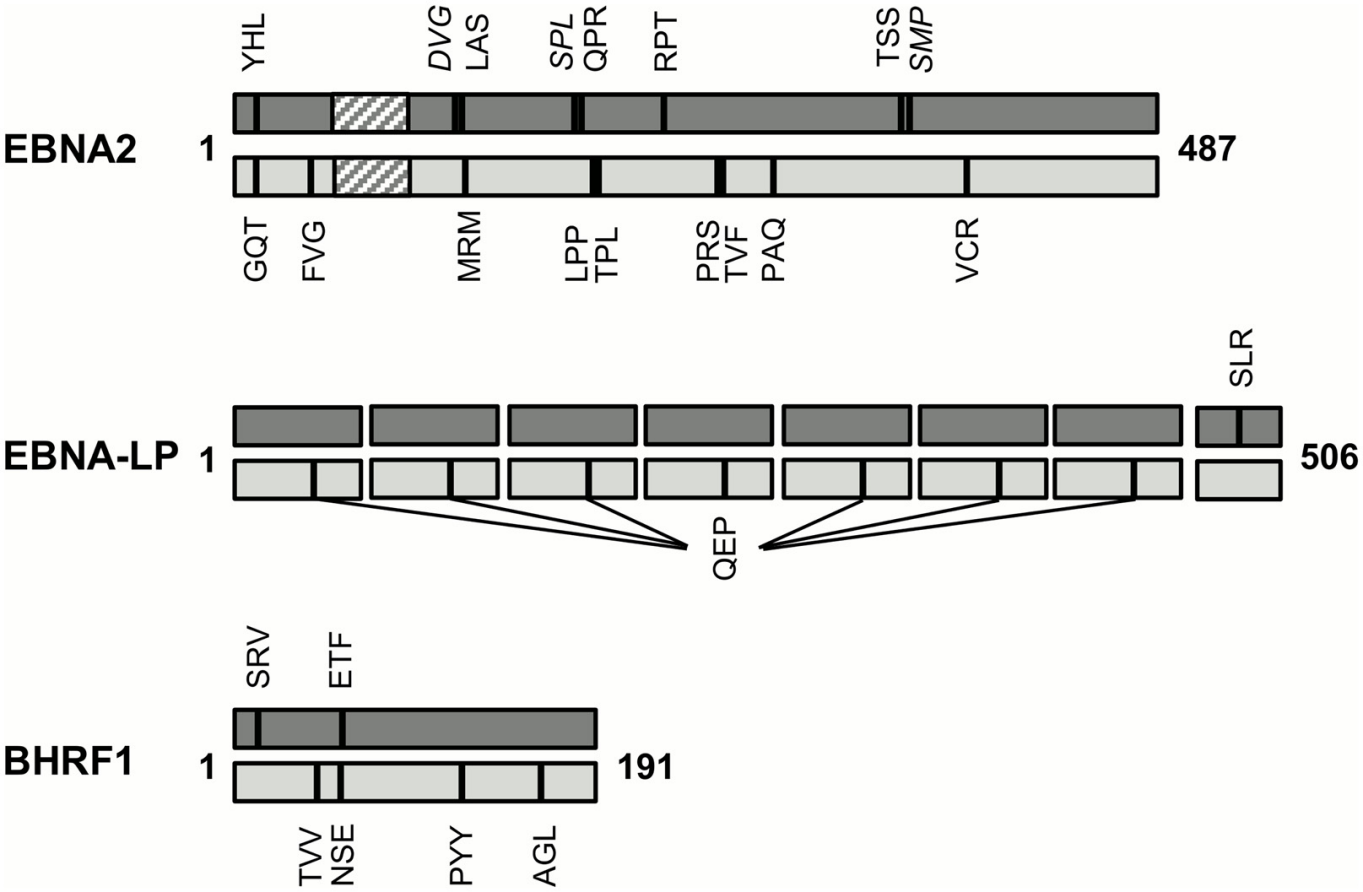
CD8 and CD4 epitope maps of EBNA-2, EBNA-LP and BHRF1. These epitope maps denote all responses identified in the present study and all previously characterised epitopes. Each protein is represented according to its relative size (B95.8 sequence; EBNA2: 487aa, EBNA-LP: (66 x 7) + 44 = 506aa, BHRF1: 191aa). The polyproline repeat domain of EBNA2 is shown as a hatched box and the seven amino terminal repeat domains of EBNA-LP as separate boxes. Epitopes (CD8 top, CD4 bottom) are illustrated as vertical bars and are identified by the first three amino acids of their sequence. CD8 responses for which the minimal epitope is not defined are shown in italics. Full details of all CD8 and CD4 epitopes relevant to the present study are given in Tables 1 and 2.

Fig 3 summarises the mapping of EBNA2, EBNA-LP and BHRF1 responses to single-epitope regions, showing the primary sequence of each protein according to its relative size and identifying the positions of all defined CD8 and CD4 epitopes within each sequence. Focussing initially on EBNA2, it can be seen that whilst both CD8 and CD4 epitopes are distributed throughout the length of the protein, there are “hotspots” where (i) CD8 or CD4 epitopes are co-localised (e.g. the two newly defined B7-restricted epitopes between amino acids 181 and 200), or (ii) CD8 and CD4 epitopes overlap (e.g. the B38-restricted YHL epitope and the DR4-restricted GQT epitope). The one EBNA-LP-derived CD8 T cell epitope lies within the short unique carboxy-terminal sequence whereas the one CD4 epitope is present in each copy of the repeat domain. For BHRF1, the two defined CD8 epitopes lie towards the amino-terminus; CD4 epitopes are distributed throughout the protein, with the one newly defined epitope encompassing the CD8 A68/ETF epitope.

### Immunodominance of EBNA2, EBNA-LP and BHRF1-specific CD8+ T cell responses

Having mapped the above CD8+ T cell epitopes using in vitro-expanded effector populations, we sought to determine (i) the relative size of such responses in PBMCs ex vivo, versus those seen against well-defined EBV epitopes (derived from latent or lytic cycle antigens [31]), and (ii) the fraction of donors expressing the relevant HLA class I allele who responded to each epitope. PBMCs were therefore screened in IFNγ Elispot assays against new and previously defined epitope peptides appropriate for each donor’s HLA class I type.

**Fig 4:**
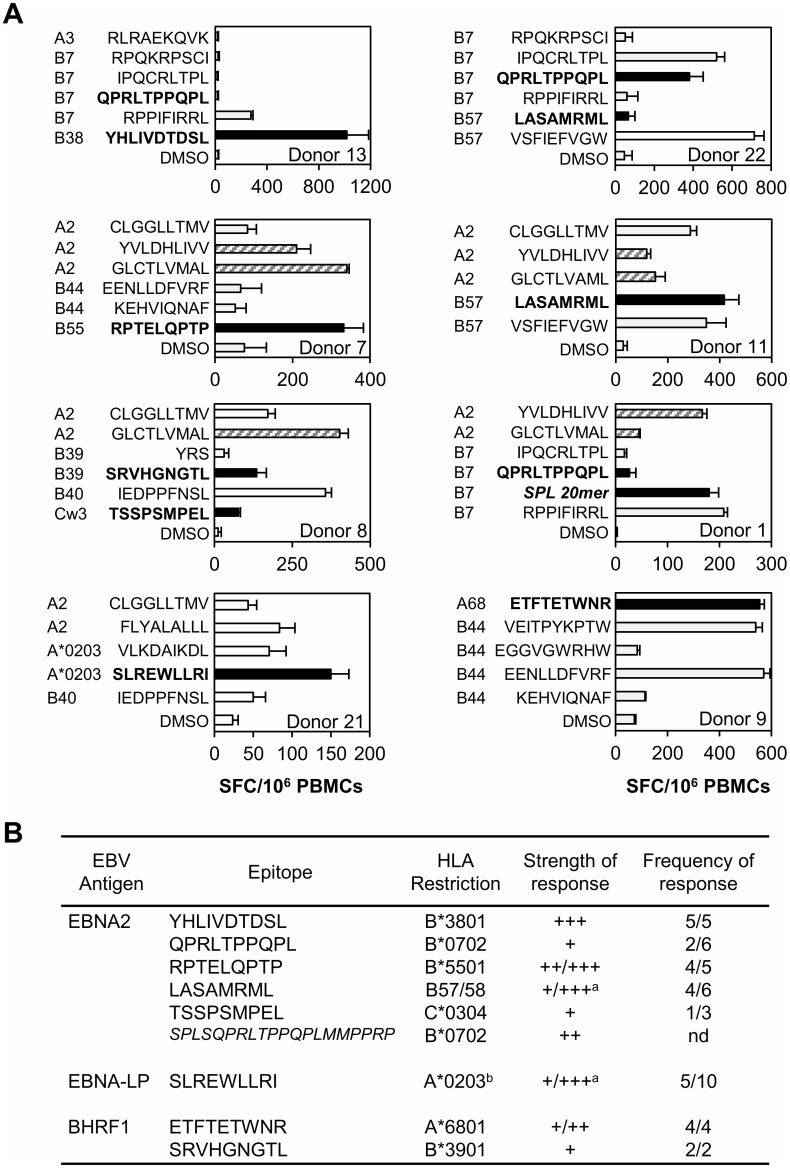
Relative magnitude and frequency of EBNA2, EBNA-LP and BHRF1-specific CD8+ T cell responses. (A) Representative IFNγ Elispot assays in which PBMCs were screened ex vivo against CD8 epitope peptides derived from EBV latent (grey bars) and lytic (hatched bars) cycle proteins, selected as appropriate for each donor’s HLA class I type, and including one or more epitopes derived from EBNA2, EBNA-LP or BHRF1 (black bars). Donors included in the screening panel are identified by number (1–20); Donors 21 and 22 were not included in the original panel. Results are expressed as the mean number of spot forming units/10^6^ PBMCs +/- SD from ≥duplicate wells. (B) Summary table of Elispot data for all available donors expressing one or more relevant class I restriction determinants for EBNA2-, EBNA-LP- or BHRF1-specific responses. Responses are categorised as immunodominant (+++, numerically greater than other measured responses), co-dominant (++, equivalent to other measured responses) or subdominant (+, smaller than other measured responses). The frequency of response represents the fraction of donors bearing the appropriate HLA class I allele who responded to the epitope. ^a^Range of response strengths between individual responsive donors. For LAS there was no correlation between expression of B57 or B58 and strength of response. nd not determined. ^b^This response was not detected in donors bearing other HLA-A2 subtypes.

Examples of results from individual donors are shown in Fig 4A and the overall results summarised in Fig 4B, with responses categorised as immunodominant (numerically greater than other measured responses), co-dominant (equivalent to other measured responses) or subdominant (smaller than other measured responses). Several of the “first wave” protein-derived epitopes induced strong responses. For example, the EBNA2-derived RPT/B*5501 response in Donor 7 (A2/B55-positive) was comparable with that to the immunodominant GLC/A2 epitope from the BMLF1 lytic cycle protein [27] (Fig 4A, second left panel). Likewise, in one of the two B57-positive subjects (Donor 11, second right panel), the EBNA2-derived LAS/B57 epitope induced the strongest response, higher even than that seen against the previously described immunodominant B57/58 epitope, VSF from EBNA3B [32]. Fig 4B not only summarises the strength of responses to EBNA2, EBNA-LP and BHRF1 epitopes but also shows the frequency with which individuals with the appropriate HLA class I allele make a detectable epitope response. Although there is wide variation in both parameters, there is a general trend for immunodominant or co-dominant responses (e.g. B*3801/YHL and B*5501/RPT) to be frequently seen in the appropriate donors, and for numerically sub-dominant responses (e.g. B7/QPR and Cw3/TSS) to be less often detectable.

### Analysis of gene expression and T cell recognition following EBV infection of B cells in vitro

As described above, EBNA2, EBNA-LP and BHRF1 are amongst the first viral proteins expressed in B cells following EBV infection and thus represent potential targets for specific T cell recognition prior to cell cycle entry, blast transformation and the establishment of latency. In subsequent experiments we therefore wanted to determine how closely T cell recognition follows the kinetics of antigen expression. The key question being addressed is whether T cell mediated control can be exercised in the initial 24–48hrs post-B cell infection, before the first cell division and prior to LMP1-mediated activation of antigen processing and presentation pathways [29,30].

**Fig 5:**
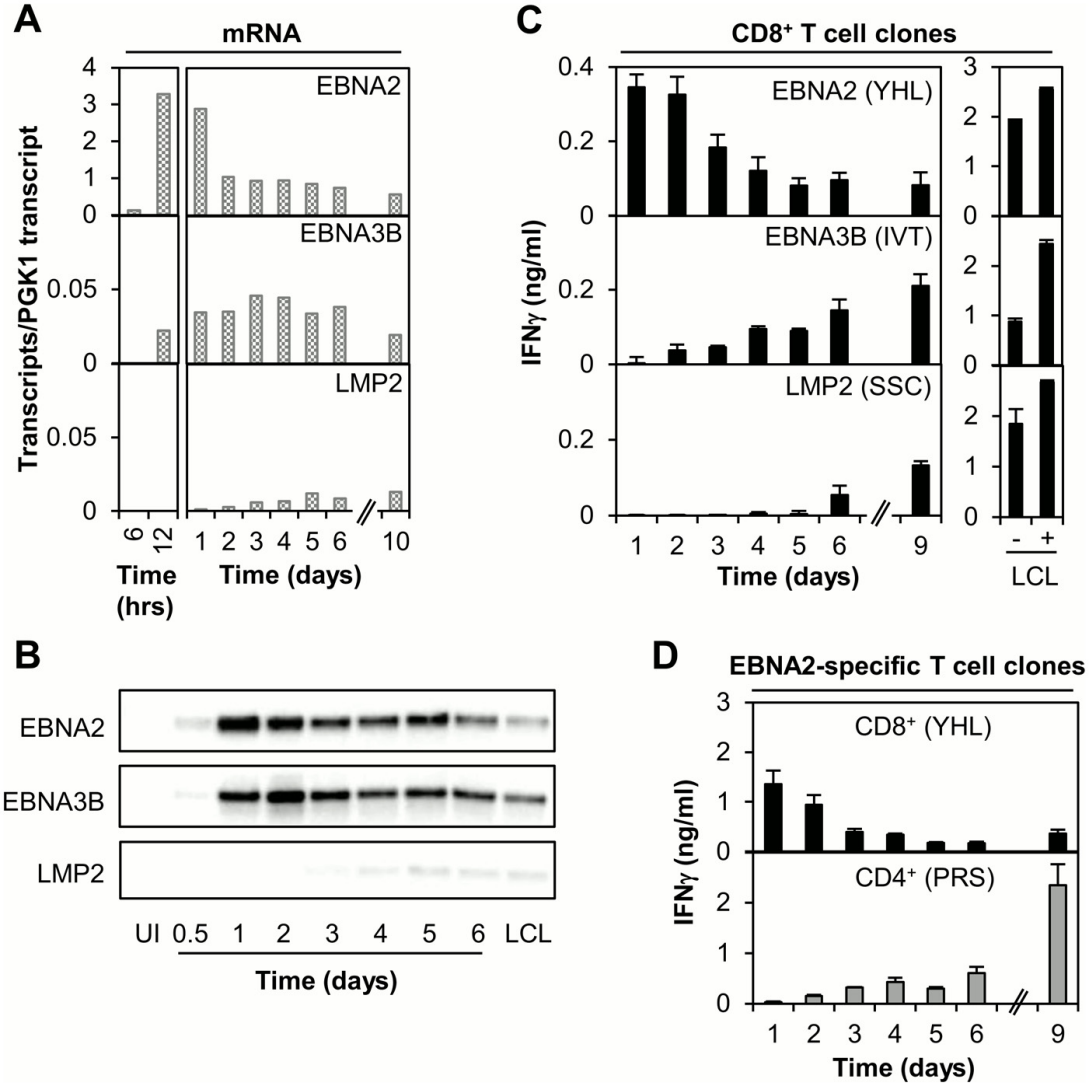
Analysis of EBNA2 expression and T cell recognition following EBV infection of B cells in vitro. (A) Analysis of gene expression using a 48:48 Dynamic Array QPCR assay to measure EBNA2, EBNA3B and LMP2 mRNA transcripts at time points between 6hrs and 10 days following infection of primary B cells with EBV (wt2089). Results are normalised to cellular PGK1 transcript levels. (B) Immunoblotting to detect expression of EBNA2, EBNA3B and LMP2 at time points between 0.5 and 6 days post EBV-infection; uninfected B cells (UI) and an established LCL were included as negative and positive controls respectively. (C) CD8+ T cell recognition of newly infected B cells. Left panels: Primary B cells (HLA-A11-, B*3801-positive) were infected with EBV (B95.8 supernatant) then co-cultured with latent antigen-specific (EBNA2: YHL/B*3801, EBNA3B: IVT/A11 LMP2: SSC/A11) T cell clones (40,000 B cells + 5000 T cells/well). Culture supernatant was harvested at the specified time points and the IFNγ concentration measured by ELISA; results are the mean of triplicate wells +/- SD. Right panels: T cell recognition of established HLA-B38 or -A11 matched LCLs -/+ cognate epitope peptide shown for reference. (D) Comparison of EBNA2-specific CD8+ versus CD4+ T cell recognition of newly infected B cells. Primary B cells (HLA-B*3801, DR7-positive) were infected with purified virus (wt2089), co-cultured with EBNA2-specific T cell clones (35,000 B cells + 2500 T cells/well) and assayed as in (C). Results for T cell co-culture assays are representative of ≥3 independent experiments using multiple effectors of different specificities.

For the analysis of gene expression, B cells isolated from buffy coat cells were exposed to EBV, cultured and then harvested at time points between 6hrs and 14 days post-infection. mRNA levels were quantified using a 48:48 Dynamic Array IFC-Gene Expression system and a plasmid standard containing a single copy of each of 45 EBV and 3 cellular amplicons [34]; this assay enables the absolute quantification of EBV transcript levels. Focusing initially on EBNA2, results for one representative experiment, here comparing the kinetics of expression of EBNA2, EBNA3B and LMP2, are shown in Fig 5A. EBNA2 transcripts were detected as early as 6hrs post-infection, peaked to a very high level within 12hrs, then fell sharply by day 2 before reaching plateau levels. EBNA3B expression was slightly delayed relative to EBNA2, becoming first detectable at 12hrs and reaching low maximal levels around days 2–3. LMP2 transcripts were first detected at very low levels on day 2 and plateaued around day 5. This temporal sequence of latent gene expression essentially accords with previously published data [34–36]; however, as recently reported [34] and apparent from the different scales used in Fig 5A, there are also major differences in the levels of these latent gene transcripts. Thus, even as early as 6hrs post-infection, EBNA2 transcript levels exceeded the maximum levels detected for both EBNA3B and LMP2. Indeed peak EBNA2 transcript levels seen at 12–24hrs were ~100-fold higher than the EBNA3B or LMP2 maximum and were still >10-fold higher than these at day 10 with the establishment of latency. To complement the mRNA analysis, protein expression was analysed by immunoblotting (Fig 5B). EBNA2 was detectable 12hrs post-infection, reaching peak levels within 24hrs. EBNA3B expression was again slightly delayed relative to EBNA2, being detectable at very low levels 12hrs post-infection and maximally around day 2; low level LMP2 expression was detectable from day 3–4 onwards.

**Fig 6:**
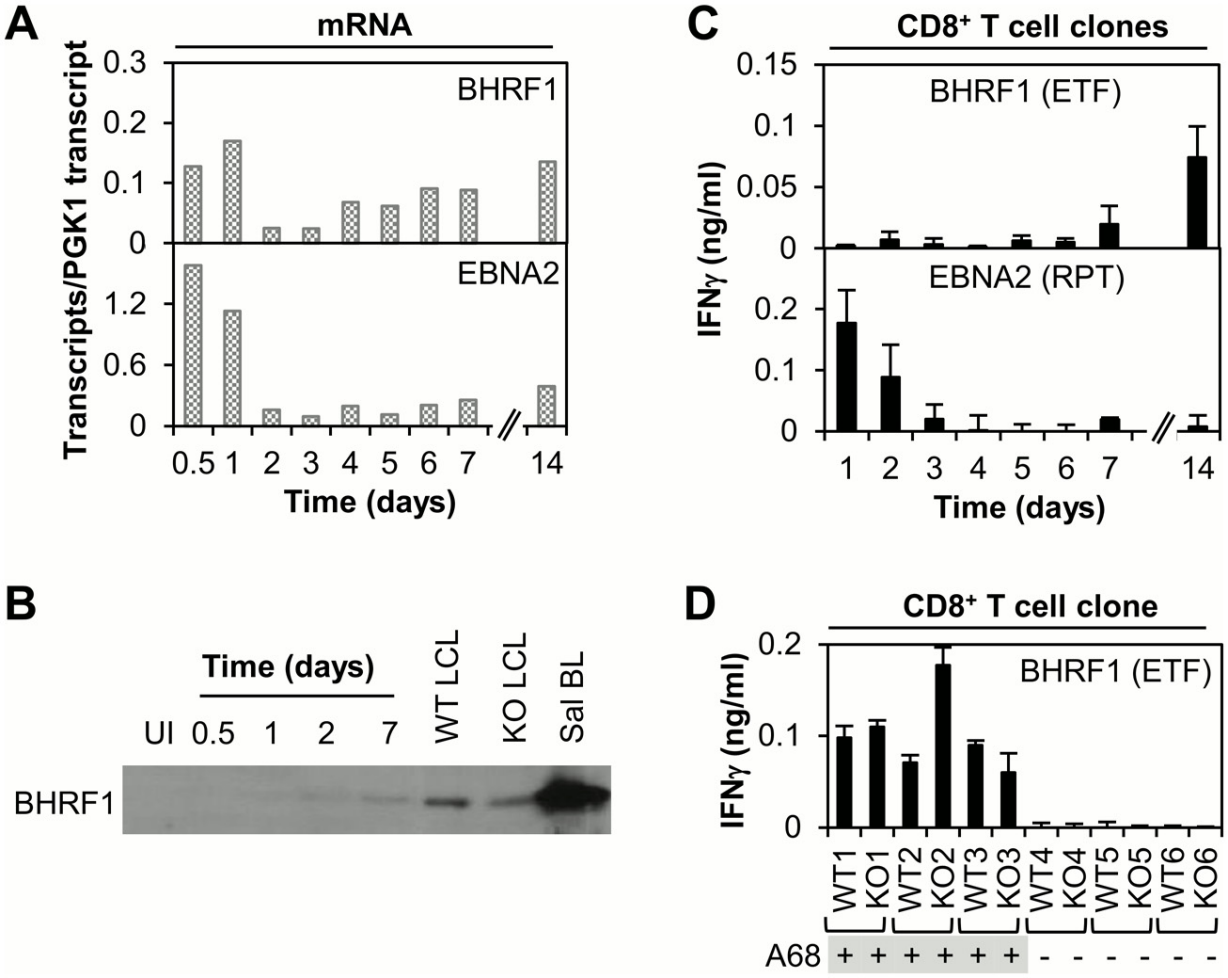
Analysis of BHRF1 expression and CD8+ T cell recognition following EBV infection of B cells in vitro. (A) Analysis of gene expression using a 48:48 Dynamic Array QPCR assay to measure BHRF (Y2-HF, latent) and EBNA2 transcripts at time points 0.5 to 14 days post-infection with EBV (wt2089). Results are expressed as in Fig 5A. (B) Immunoblotting to detect expression of BHRF1 at time points between 0.5 and 7 days post EBV-infection; uninfected B cells (UI), established wild-type (WT) and BZLF1-KO LCLs and a BL cell line (Sal) were included as controls. (C) CD8+ T cell recognition of newly infected B cells. Primary B cells (HLA-A68, B*5501-positive) were infected with EBV (wt2089) then co-cultured with BHRF1 (ETF/A68) and EBNA2 (RPT/B*5501)-specific CD8+ T cell clones (40,000 B cells + 2500 T cells/well). Supernatant was harvested at the specified time points and the IFNγ concentration measured by ELISA; results are the mean of triplicate wells +/- SD. (D) BHRF1 expression during latency. BHRF1 (ETF)-specific CD8+ T cell clones were screened against a panel of HLA-matched (A68-positive) and mismatched LCL pairs; each LCL pair included the WT and BZLF1-KO LCL. IFNγ ELISA, results are shown for one representative clone. Results for T cell assays are representative of ≥2 independent assays using multiple effectors of each specificity.

For T cell recognition assays primary B cells were isolated from healthy adult donor PBMCs of appropriate HLA class I/II type, infected with EBV and then co-cultured with specific T cell clones in medium supplemented with interleukin-2 (IL-2). Culture supernatant was sampled at regular intervals between days 1 to 14 post-infection and the IFNγ concentration quantified by ELISA as a measure of T cell recognition. Illustrative results for one experiment, evaluating CD8+ T cell recognition of EBNA2, EBNA3B and LMP2 are shown in Fig 5C (left panels); recognition of established LCLs is shown for reference (right panels). CD8+ T cells specific for the EBNA2-derived YHL epitope recognised EBV-infected B cells at the earliest assay time point, day 1 post-infection; similar results were obtained using CD8+ clones specific for a second EBNA2-derived epitope (RPT, Fig 6C). By contrast, recognition of EBNA3B (represented by the IVT epitope) was first detectable from day 2 post-infection and increased thereafter; CD8+ T cell clones specific for epitopes derived from EBNA3A, 3C and EBNA1 gave similar results to EBNA3B (S2A Fig). Recognition of LMP2 (represented by the SSC epitope) was further delayed, only reaching detectable levels on day 6. Absolute levels of IFNγ production varied between individual new-infection experiments (for example compare top panels of Fig 5C and 5D), and were generally lower than those induced by established LCLs. However, the pattern of CD8+ T cell recognition for individual latent proteins was consistent and broadly correlates with the kinetics of antigen expression. It is interesting to compare such recognition kinetics with those seen using CD4+ T cell clones specific for these antigens, for example EBNA2. Though EBNA2 is highly expressed within the first 2 days post-infection, CD4+ T cell recognition of this protein is essentially undetectable at such early times and appears only gradually thereafter (Fig 5D).

EBNA-LP is also known to be highly expressed at the protein level in newly-infected B cells [36,37], but comparable experiments with CD8+ effectors against the EBNA-LP-derived SLR epitope could not be conducted because of limited access to PBMCs from A*0203-positive subjects. However, we were interested to extend this work to study the kinetics of BHRF1-specific CD8+ T cell recognition in relation to expression of this protein during the B cell transformation process. Note that BHRF1 is traditionally considered to be an early lytic cycle antigen, but can also be expressed as a latent gene product from transcripts initiating from Wp, the promoter that initially drives both EBNA2 and EBNA-LP expression [36]. Fig 6A shows the expression levels of such BHRF1 and EBNA2 mRNAs as determined by 48:48 Dynamic Array QPCR assay in one representative experiment. Latent BHRF1 transcripts were maximally detected on day 1, then temporarily declined, recovering slightly from day 4 onwards. Though absolute mRNA values differed between individual transformation experiments, peak BHRF1 transcript levels were usually ~10-fold lower than those for EBNA2. Furthermore at the protein level, immunoblotting showed that (unlike EBNA2) the BHRF1 protein was barely detectable at day 1 and increased only gradually with time. Even at 7 days the protein had not achieved the stable levels that can be detected in established LCLs, whether they carry replication-competent (wild-type) or replication-defective (BZLF1-knockout) virus (Fig 6B). Results for one representative T cell assay, comparing recognition of newly infected B cells by CD8+ effectors specific for BHRF1 and EBNA2, are illustrated in Fig 6C. Here, T cell clones specific for the EBNA2-derived RPT epitope recognised EBV-infected B cells from day 1 (lower panel). In contrast, BHRF1-specific T cells showed no recognition at early time points; recognition only becoming detectable, albeit at low levels, by day 14 (upper panel). This late level of recognition is equivalent to that observed for established LCLs, again whether carrying wild-type or BZLF1-knockout virus (Fig 6D), suggesting that such recognition is predominantly mediated by BHRF1 expressed as a latent cycle antigen. We infer that BHRF1, in contrast to EBNA2, is a poor target antigen for CD8+ T cell recognition early post-B cell infection, likely reflecting the low level of BHRF1 protein expression seen at these times.

**Fig 7:**
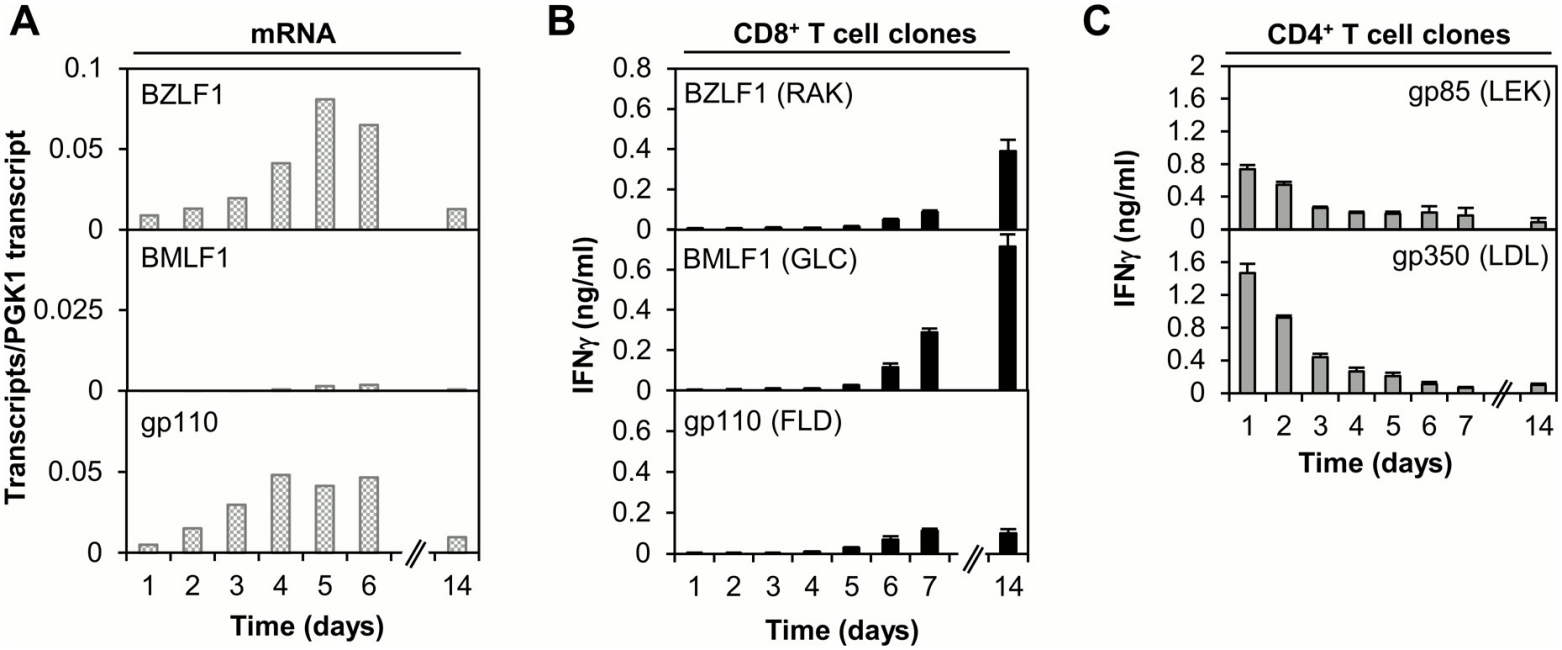
Analysis of lytic cycle gene expression and T cell recognition following EBV infection of B cells in vitro. (A) Analysis of gene expression using a 48:48 Dynamic Array QPCR assay to measure lytic transcripts (BZLF1, BMLF1 and BALF4 (gp110)) at time points between 1 and 14 days post-infection with EBV (wt2089). Results are expressed as in Fig 5A. (B) CD8+ T cell recognition of newly infected B cells. Primary B cells (HLA-A2, B8-positive were infected with EBV (B95.8 supernatant) then co-cultured with lytic antigen-specific (IE: BZLF1 RAK/B8, E: BMLF1 GLC/A2, L: gp110 FLD/A2) CD8+ T cell clones (40,000 B cells + 3000 T cells/well). Supernatant was harvested at the specified time points and the IFNγ concentration measured by ELISA; results are the mean of triplicate wells +/- SD. (C) CD4+ T cell recognition of newly inflected B cells. Primary B cells (HLA-DR15, DR51-positive) were infected with EBV (purified wt2089) then co-cultured with L lytic antigen-specific (gp85 LEK/DR51, gp350 LDL/DR15) CD4+ T cell clones (40,000B cells + 5000 T cells/well) and assayed as in (B). Results for T cell co-culture assays are representative of ≥3 independent experiments using multiple effectors of different specificities.

In a next series of experiments we analysed the kinetics of T cell recognition of lytic cycle antigens. It has been reported previously that IE/E lytic cycle proteins, transiently expressed in newly infected B cells from viral mRNAs carried within mature virus particles, constitute target antigens for early CD8+ T cell recognition [24–26]. We sought to replicate these results using our experimental protocols. Fig 7A shows results for one 48:48 Dynamic Array QPCR assay, measuring transcript levels for representative IE (BZLF1), E (BMLF1) and L (BALF4/gp110) lytic cycle antigens. All lytic cycle transcripts were detected at very low levels throughout the 14 day time course, although a small peak of expression was observed around day 5. Of note, transcript levels on day 1 post-infection were >100-fold lower than those for EBNA2 (compare Fig 7A with Figs 5A and 6A). The same panel of lytic cycle proteins was assessed as target antigens for CD8+ T cell recognition on days 1–14 post-EBV infection of B cells; results for one representative experiment are shown in Fig 7B. T cell recognition was undetectable at early time points, becoming measurable around days 6–7, and then increasing over time to day 14. This pattern of recognition was reproduced in experiments using T cell clones specific for four different IE/E lytic antigen-derived CD8 epitopes. Absolute levels of T cell recognition at late time points differed between separate experiments, presumably reflecting variable numbers of B cells spontaneously entering lytic cycle at these later times.

**Fig 8:**
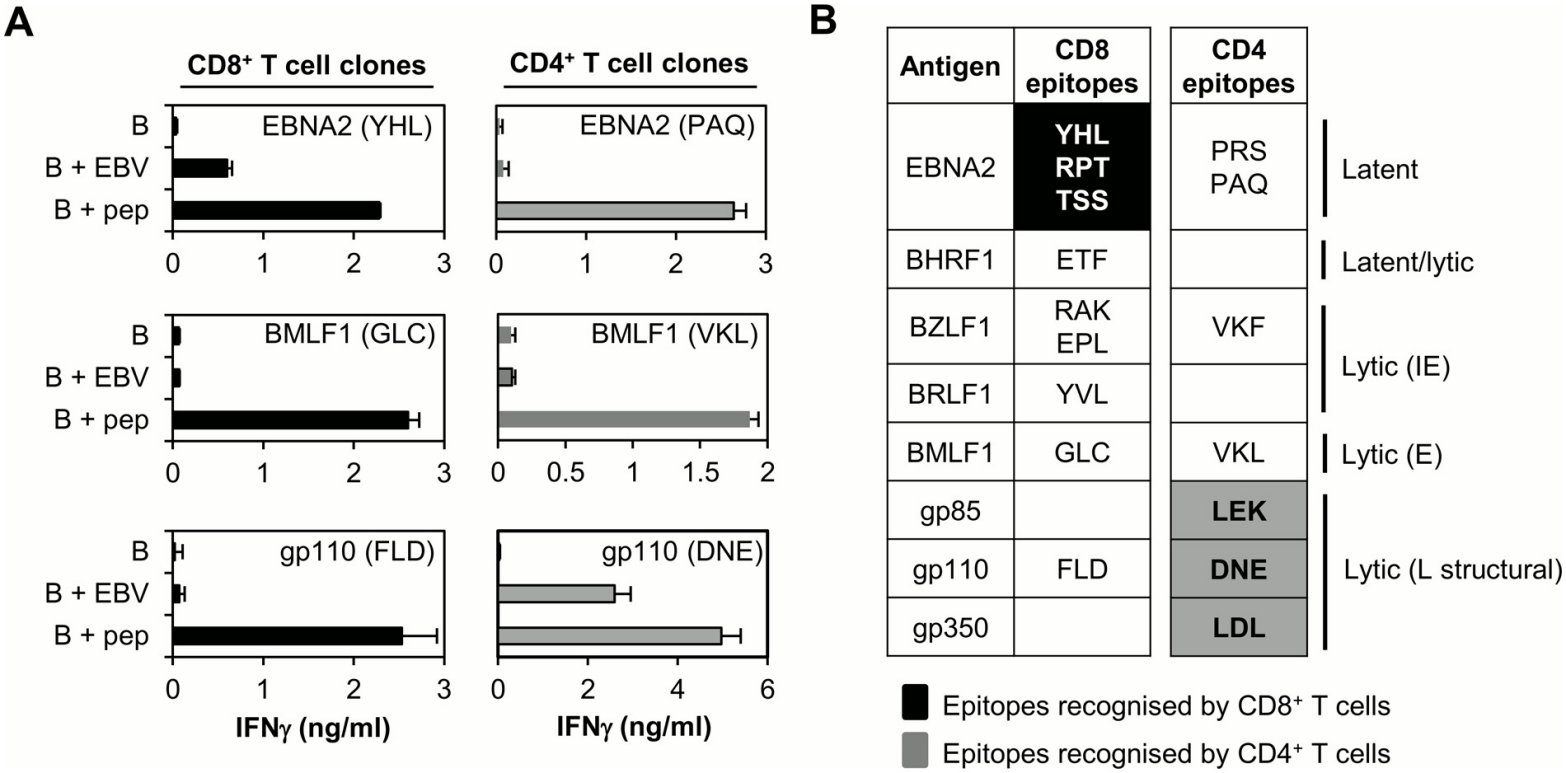
CD8+ and CD4+ T cell recognition of B cells 24hrs post-EBV infection. (A) Representative plots showing CD8+ (left panels, black) and CD4+ (right panels, grey) T cell recognition of primary B cells 24hrs post-EBV infection (B + EBV) by effectors specific for EBNA2 (latent, top), BMLF1 (E lytic, middle) and gp110 (L lytic structural, bottom). Uninfected B cells (B) and epitope-peptide loaded B cells (B + pep) are included as negative and positive controls respectively. (B) Summary of T cell recognition data for all epitopes included in the study and derived from “first wave” (EBNA2, BHRF1) or IE (BZLF1, BRLF1) E (BMLF1) and L (gp85, gp110, gp350) lytic cycle antigens. Epitopes recognised by CD8+ and CD4+ effectors at the 24hr time-point are highlighted in black or grey respectively; epitopes on a white background are not recognised by CD8+ or CD4+ effectors within 24hrs post-EBV infection.

Lytic cycle proteins therefore appear to constitute poor targets for immediate recognition by CD8+ T cells following EBV infection of B cells. However, protein components of the viral envelope and capsid may be efficiently processed via the class II pathway for immediate recognition by CD4+ T cells. Indeed, it has previously been shown that glycoprotein-specific CD4+ T cells can recognise B cells at early time-points post-EBV infection [17]. As comparators in the CD8 recognition experiments, we likewise assayed for CD4+ T cell recognition of newly infected B cells using effectors specific for the viral glycoproteins gp85, gp110 and gp350; representative results are shown in Fig 7C. CD4+ T cell clones specific for epitopes derived from gp85 and gp350 recognised EBV-infected B cells at the earliest assay time point, 1 day post-infection; similar kinetics were seen in other experiments probing with gp110-specific CD4+ effectors (Fig 8A, bottom right panel).

Fig 8 summarises all the data for CD8+ and CD4+ T cell recognition of primary B cells 24hrs post-EBV infection. EBNA2 is efficiently processed and presented for CD8+ T cell recognition at this time, whereas BHRF1 and IE/E lytic cycle antigens appear not to be. Furthermore, CD8+ T cells specific for gp110 also failed to detect recently-infected B cells, arguing against cross-presentation of viral envelope glycoproteins to CD8+ effectors. For CD4+ T cells the pattern of results is quite different. CD4+ T cell clones specific for epitopes derived from latent, or IE/E lytic cycle proteins recognised B cells loaded with the appropriate synthetic peptide, but failed to recognise EBV-infected B cells. However, CD4+ T cells specific for epitopes derived from glycoproteins that are components of the incoming virion (L lytic) recognise newly infected B cells strongly within 24hrs.

### CD8+ T cell inhibition of B cell transformation and LCL outgrowth

**Fig 9:**
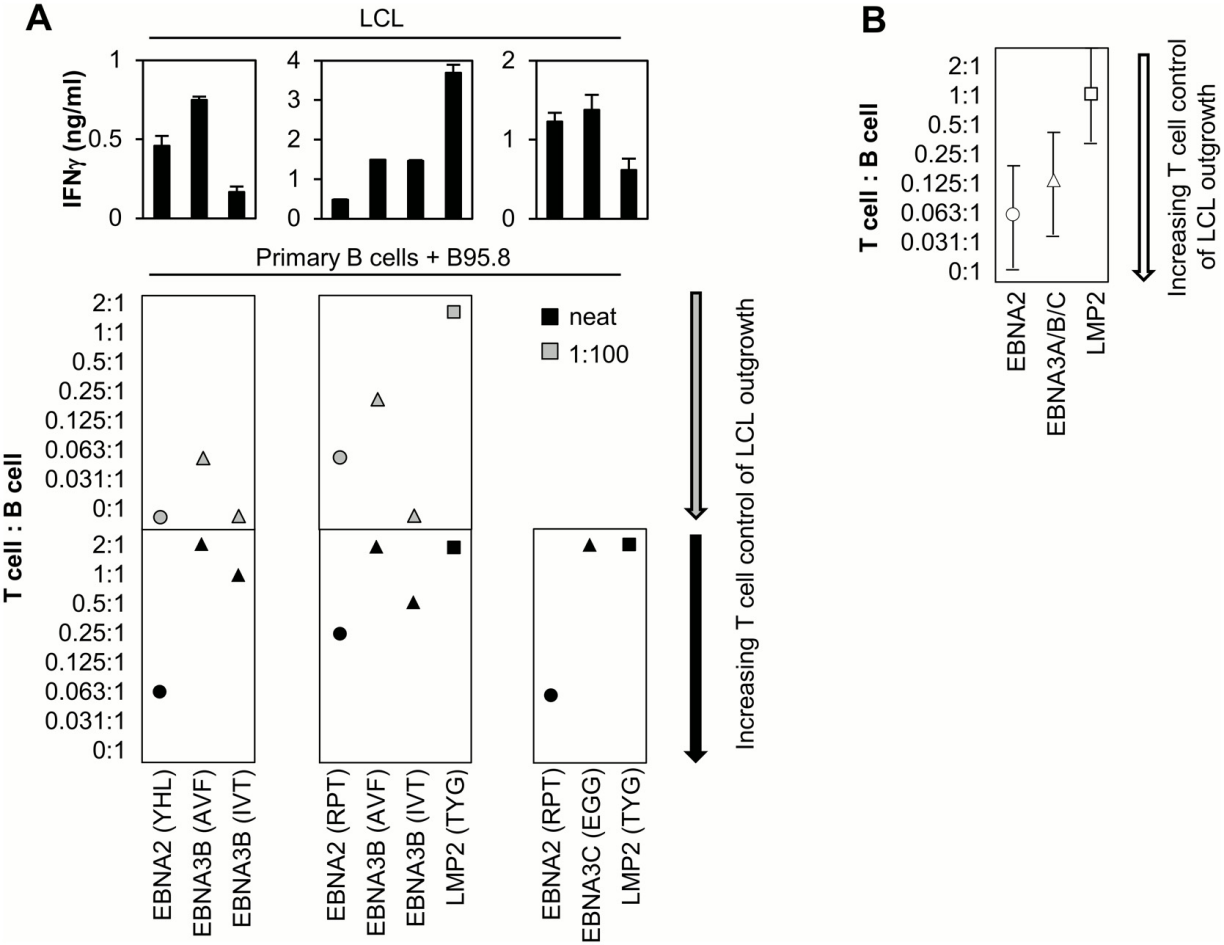
CD8+ T cell inhibition of B cell transformation and LCL outgrowth. (A) Representative results from three experiments where primary B cells were infected with B95.8 supernatant, either neat (bottom panels) or diluted 1:100 (middle panels) and co-cultured with EBNA2-, EBNA3B/C- or LMP2-specific CD8+ T cell clones at effector to target ratios between 2:1 and 0:1. Results (shown for one effector population of each specificity/experiment) are expressed as the minimum T cell seeding required to completely inhibit B cell transformation and LCL outgrowth, scored visually at ~4 weeks. T cell recognition of an established LCL from the B cell donor is included for reference (top panels). (B) Combined results (mean +/- SD) for all EBNA2 (YHL/RPT), EBNA3 (RPP/AVF/IVT/EGG) and LMP2 (TYG)-specific effectors from six independent experiments.

A final set of experiments compared the ability of EBNA2 versus other latent antigen-specific CD8+ T cell clones to inhibit B cell transformation and LCL outgrowth following EBV infection in vitro. Primary B cells were isolated from PBMCs, infected with EBV at two virus doses (neat and 1:100), and then both infected populations co-cultured with specific CD8+ T cells at a range of effector:target ratios (between 2:1 and 0:1). Cultures were maintained by weekly re-feeding and scored visually for LCL outgrowth at ~4 weeks. In each case the B cell donor was selected to allow HLA class I matching of the same infected B cells with T cell clones against the different latent antigens. Representative results from three such experiments are illustrated in Fig 9A, here comparing inhibition of LCL outgrowth by CD8+ effectors specific for epitopes derived from EBNA2 (YHL and RPT), EBNA3B (IVT and AVF) and LMP2 (TYG); recognition of an established LCL from the B cell donor is included for reference. Combined results for all experiments are shown in Fig 9B. In this experimental setting EBNA2-specific T cells appeared to be as efficient, and often more efficient, than EBNA3-specific effectors in their ability to control B cell transformation and LCL outgrowth, especially at higher virus doses; LMP2-specific effectors, at least as represented by TYG-specific T cell clones, were reproducibly poorer in this regard.

## Discussion

An effective prophylactic EBV vaccine should aim to prevent, or at least limit colonisation of the B cell system, the process that is essential for virus persistence and central to the development of most, if not all, EBV-associated disease. An ideal vaccine might induce both neutralising antibodies to reduce levels of infection and T cell responses to target B cells that do become infected. Proteins that are expressed in the very early phase of B cell transformation, including EBNA2, EBNA-LP and BHRF1, constitute potential vaccine immunogens for the induction of CD8+ T cell responses. Here we have (i) determined to what extent natural EBV infection elicits CD8 responses to these “first wave” proteins, including identification of target epitopes/HLA restricting alleles and assessment of relative immunodominance; (ii) assayed the ability of such T cells to recognise B cells expressing these proteins in the very early phase of infection, prior to cell cycle entry; (iii) compared such recognition with that shown by CD8+ T cells against other potential targets of early detection i.e. IE/E lytic antigens, that may be expressed from mRNAs contained within the virion [22–26], and virus structural proteins delivered into B cells upon infection; and (iv) assessed the ability of such effectors to inhibit EBV-induced B cell transformation and LCL outgrowth.

To characterise the immunogenicity of “first wave” proteins, PBMCs from healthy seropositive donors were stimulated with peptides representing EBNA2, EBNA-LP or BHRF1, and T cell responses assessed by cytokine production following a brief period of in vitro expansion to maximise detection sensitivity. The relative magnitude and frequency of “first wave” responses were then compared with previously defined latent/lytic cycle epitope responses using ex vivo assays. This work clearly identified EBNA2 as a strong CD8 immunogen in certain HLA contexts, for example B*3801 and B*5501. Likewise EBNA-LP and BHRF1, although relatively infrequently targeted by CD8+ T cells among donors in our panel, both contain epitopes that are immuno-/co-dominant in the appropriate HLA context. This mirrors the situation with respect to another latent protein, EBNA1, which despite the influence of its internal glycine-alanine repeat domain elicits strong CD8 responses in the context of particular HLA alleles such as HLA-B*3501 and -B53 [38]. It would therefore appear that all endogenously expressed EBV latent antigens (with the possible exception of LMP1 [39,40]) are accessible to the MHC I antigen processing pathway and can induce strong CD8 responses at least in a subset of individuals. We infer that the marked immunodominance of the EBNA3A,3B,3C family noted in early studies [41,42] can, at least in part, be explained by two contributory factors. One is the sheer size of the EBNA3 proteins; thus EBNA3A, 3B and 3C together provide around 60% of the total unique amino acid sequence content of all the latent proteins, compared to ~9% for EBNA2, ~2% for EBNA-LP and ~4% for BHRF1. Another is the fact that most studies of EBV-induced CD8+ T cell responses to date have involved Caucasian subjects and some of the strongest responses restricted through common Caucasian HLA I alleles (e.g. HLA-B7, -B8 and -B44) happen to involve EBNA3-derived epitopes. By comparison, CD8+ T cell responses to “first wave” proteins that are restricted through common Caucasian class I types are often subdominant and immunodominant responses are restricted through less common alleles.

We also note that, with one exception, all of the novel CD8+ T cell epitopes described herein are restricted through HLA-A or -B alleles. Indeed the exception, the HLA-C*0304-restricted TSS epitope, is only the second EBV latent antigen-derived response found to be restricted through an HLA-C allele [43]; by contrast there are at least six known EBV lytic antigen-derived responses presented by HLA-C alleles [20,21,31]. The apparently higher incidence of HLA-C-restricted responses to lytic cycle antigens may reflect the influence of the immune-evasin BILF1, whose expression during early lytic cycle selectively down regulates antigen presentation via HLA-A and -B, but not HLA-C [44].

Whilst the main focus of this study was CD8+ T cell responses, CD4+ responses to the three “first wave” antigens were analysed in parallel. In agreement with previously published work [45], EBNA2 was found to be a frequent target antigen for CD4+ T cell responses (17/19 donors responsive). Although there are at least nine defined EBNA2-derived CD4 epitopes (Fig 3), a significant part of this response is focused on a single epitope (PRS), which was detected in more than half of subjects, and is presented through multiple common class II allotypes including DR7 and DR52b [45]. In the present work, a single novel CD4 epitope was identified from EBNA-LP (QEP) and detected in up to 40% of donors. This response was not fully characterised but, like the PRS response, appears to be promiscuously presented, being detectable in donors of disparate class II type. The QEP epitope derives from the repeat region of EBNA-LP; therefore multiple copies are present within the protein, which may also contribute to the observed immunodominance.

Our next key objective was to examine T cell recognition of “first wave” proteins during the process of B cell transformation, using well-established target antigens such as EBNA3B and LMP2 as comparators. To achieve this we used a short-term co-culture protocol which (i) minimises the number of B cells required, thus allowing the analysis of T cell responses restricted through less common HLA class I types, as well as combinations of multiple class I/II alleles; and (ii) removes counting errors inherent within the set-up of individual assays on a daily basis. One potential disadvantage of the assay design is that T cells of certain specificities (e.g. LMP2) are in co-culture for several days before their cognate antigen is expressed. However, since T cell clones are routinely maintained with only periodic re-stimulation, we consider it unlikely that this would result in effectors becoming unresponsive within the assay time-frame. Relatively high virus doses were used in these assays to ensure that a majority of B cells were infected and thus had the potential to express genes/proteins of interest and to function as T cell targets. Since the mean number of EBV genomes acquired per cell remains low [46], this increases the sensitivity of detection, but does not disproportionately increase gene expression/de novo protein synthesis on a per cell basis. IFNγ production was used as the read out for specific T cell recognition, as we find this to be the most sensitive cytokine in the EBV system. To determine how closely T cell recognition follows the kinetics of antigen expression, co-culture assays were combined with new assays quantitating latent/lytic transcript levels and with immunoblotting to assess the appearance of latent proteins. Our previous measurements of EBV gene expression using conventional QRT-PCR assays [35,47,48] delineated expression patterns for individual transcripts, but could not accurately measure the abundance of different transcripts relative to each other because of using different standards. The plasmid standard, containing a single copy of 45 EBV and 3 cellular control amplicons, used here in the 48:48 Dynamic Array QPCR assay allows absolute quantitation, thus enabling the direct comparison of transcript levels for different genes at a single time point.

Using these assays we found EBNA2 to be well recognised by CD8+ effectors very early post-infection; levels of CD8+ T cell recognition at 24hrs were higher than for all subsequent time points mirroring the peak of Wp driven EBNA2 transcription and closely correlating with new EBNA2 protein synthesis. Thus even minimally activated B cells are able to process antigen for CD8 epitope display, prior to the first cell division and in advance of LMP1 expression (detectable from ~day 3/4; [36]) and up-regulation of antigen presentation pathways [29,30]. The independence of CD8+ T cell recognition from cell cycle entry was further emphasised in experiments using a recombinant virus deleted for EBNA2. B cells infected with EBNA2-KO virus do not enter cell cycle, but overexpress EBNA1 and the EBNA3 proteins from day 1 post-infection [48], and indeed we found that these B cells are well recognised by EBNA1- and EBNA3-specific CD8+ T cell clones at these atypically early times (S2B Fig). However, in experiments using wild-type virus, CD8+ T cells specific for EBNA1 and the EBNA3A, 3B, 3C antigens only recognised newly infected B cells at low levels from ~2 days post-infection and this recognition increased with time (Fig 5C and S2A Fig). The apparent delay in EBNA3-specific T cell recognition compared with mRNA expression in the present experiments may relate to the surprisingly low transcript levels for these proteins (Fig 5A). Low expression might lead to increased dependence on B cell activation and the up-regulation of antigen presentation pathways and/or increased susceptibility to the effects of co-expressed immune-evasins [25,26]. By contrast, the much higher expression levels of EBNA2 might counteract any such immune-evasion strategies, allowing sufficient representation of EBNA2-derived epitopes on the B cell surface to mediate specific CD8+ T cell recognition. Note that, for CD8+ T cells of all specificities, recognition of established LCLs is superior to that of newly-infected B cells, suggesting that antigen presenting function becomes optimal in the fully growth-transformed state. As a measure of the biological effectiveness of early B cell recognition by EBNA2-specific effectors we compared their ability to inhibit B cell transformation and LCL outgrowth with that of other latent antigen-specific CD8+ T cells. Importantly, the hierarchy of different antigen specificities in their control of early B cell outgrowth (i.e. EBNA2 ≥ EBNA3 > LMP2; Fig 9) closely correlates with the level and timing of target antigen expression in the initial days post-infection.

In contrast to the above work with EBNA2-specific CD8+ T cell clones, there was no detectable BHRF1-specific recognition at early time points post-infection, even though CD8+ effectors had high functional avidity as measured by peptide titration assays (S3 Fig). Instead, the T cell data show slow accruement of BHRF1-specific recognition during the transformation process. At later time points some fraction of this may result from small numbers of B cells entering lytic cycle, however, most appears to reflect the low but progressive expression of BHRF1 as a latent antigen. Thus LCLs made with a BZLF1-KO virus continue to express the protein and are recognised by BHRF1-specific CD8+ effectors at similar levels to WT-LCLs. We infer that low levels of protein expression at very early time-points, although able to mediate essential anti-apoptotic effects [22,36], are insufficient to allow detectable display of BHRF1-derived epitopes at the B cell surface. Unfortunately parallel experiments using EBNA-LP-specific CD8+ effectors could not be carried out due to limited availability of A*0203-positive B cells. However, we would predict similar results to those seen for EBNA2 for two reasons: (i) EBNA-LP transcripts like those for EBNA2 initially derive from the Wp promoter which reaches peak activity ~12hrs post-infection and then declines [36], and (ii) EBNA-LP protein is highly expressed by day 1 post-infection at levels easily detectable by immunoblotting [36,37].

An alternative antigen source for early CD8+ T cell recognition derives from IE/E lytic cycle gene products whose reported expression immediately post-infection [22–25] has recently been attributed to the translation of viral mRNAs transduced during B cell infection [26]. This is of particular interest because several such proteins constitute immunodominant targets for CD8 responses [20,27]. However, in our assays we did not detect early CD8+ T cell recognition of IE/E lytic antigens (including BZLF1, BRLF1 and BMLF1), despite using high avidity clones that showed good recognition of these antigens when expressed in B cells during lytic replication. The absence of T cell recognition at early time points accords with mRNA quantitation results where the relevant transcripts are detected at very low levels, if at all. Single cell staining suggests that the small peak of BZLF1 expression detected around Day 5 represents a minority of B cells entering lytic cycle, which fraction may be insufficient to mediate T cell recognition at this time. Expression of IE/E lytic cycle proteins immediately post-infection will have opposing effects on CD8 recognition: proteins may be processed and presented via the class I pathway and behave as T cell targets; alternatively, those with immune-evasion functions may inhibit recognition of self and any co-expressed antigens. Thus in a previous study [25] CD8+ effectors recognised B cells infected with a recombinant virus deleted for BNLF2a (an inhibitor of the transporter associated with antigen processing [49]) at day 1 post-infection, but not wild-type virus-infected B cells; however, none of the “first wave” proteins were investigated as target antigens.

The final potential source of CD8 epitopes for early T cell recognition considered here comprises of viral structural proteins. Opportunities to study this were limited because late antigen-specific CD8+ T cells are rarely detectable in healthy virus carriers, and until recently only a limited number of epitopes were defined [20,31]. Cross-presentation of virion component proteins for CD8+ T recognition was not observed for the single gp110-derived epitope tested. However, other virus structural antigens such as capsid proteins, that naturally enter the cytosol during transport to the nucleus, may differentially access the class I processing pathway. Work in our laboratory has recently identified several new responses to L antigens including tegument and capsid as well as envelope proteins [21]; the potential of such antigens to be cross-presented for CD8+ T cell recognition remains to be determined.

Immune correlates for protection from EBV-associated disease are not fully understood. To date, vaccines that have shown promise in clinical trials for the prevention of IM have focused on gp350 [12,13], the major target of the neutralising antibody response. However it is not known to what extent such vaccines exert their effects through induction of neutralising antibodies and/or T cell-mediated immunity. CD4+ T cells specific for gp350 (and other virion glycoproteins) are capable of recognising B cells at very early time-points post-EBV infection in vitro ([17] and Figs 7 and 8) and may well contribute towards vaccine-induced protection. Vaccine efficacy might be further improved by eliciting CD8+ T cell responses capable of recognising and killing recently-infected B cells at the very early stages of virus-induced transformation. Our data suggest that at least one “first wave” protein EBNA2, and possibly a second, EBNA-LP, have the potential to serve as such candidate immunogens. This study focussed for the most part on Caucasian HLA types; in the future it would be interesting to look for CD8+ T cell responses restricted through class I types present in non-Caucasian populations (like the A*0203-restricted EBNA-LP response) which could increase vaccine range.

## Materials and Methods

### Ethics statement

All experiments were approved by the West Midlands—Black Country NRES Committee (07/Q2702/24). All donors provided written informed consent for the collection of blood samples and subsequent analysis.

### Peptides

Overlapping peptides (20mers overlapping by 15aa or 15mers overlapping by 10aa) spanning the complete unique sequences of EBNA2, EBNA-LP and BHRF1 were dissolved in DMSO. Peptides were combined into pools comprising 5–6 adjacent peptides, each at a concentration of 100μg/ml. Overlapping and minimal epitope peptides were synthesised by Alta Bioscience, Abingdon, UK or Peptide 2.0, Virginia, USA.

### Generation of polyclonal T cell populations

PBMCs were isolated from whole blood by density gradient centrifugation and incubated with peptide pools at a final concentration of 1μg/ml in RPMI containing 8% human serum (HuS; TCS Biosciences Ltd,) for 1½ hours. Peptide loaded PBMCs were washed, and then plated out in 24 well plates at a maximum density of 5 million cells/well in RPMI/HuS supplemented with 10ng/ml IL-7 (Peprotech). IL-2 (Novartis Pharma) was added on day 3 at a final concentration of 20IU/ml. Polyclonal T cell populations were screened for peptide specificity after 7 days of culture.

### Screening polyclonal T cell populations for peptide specificity

Dependent on available cell numbers total and/or CD4-positive as well as CD4-depleted polyclonal T cell populations were screened against peptide sub-pools in duplicate. CD4-positive T cells were isolated using Dynabeads according to the manufacturer’s instructions. The average purity of T cell populations, where sampled, was: CD4-positive <1% CD8+; CD4-depleted <8% CD4+. 100μl of T cells were plated out in 96V-well plates at cell densities between 50,000 and 175,000 cells/well; 100μl of peptides were then added at a final concentration of 1μg/ml. Co-cultures were incubated at 37°C overnight. Specific peptide recognition was measured by IFNγ ELISA (Perbio) according to the manufacturer’s protocol.

### Generation of T cell clones

T cell clones were obtained by limiting dilution from peptide-stimulated polyclonal populations or from polyclonal cultures stimulated with the autologous LCL, as previously described [50]. Briefly, T cells were plated out at 3 or 0.3 cells/well in 96U well plates with 10^5^ PHA-treated irradiated allogeneic PBMCs and 10^4^ irradiated autologous LCL cells or 10ng/ml OKT3 monoclonal antibody. All growing wells were screened for recognition of the appropriate peptide by IFNγ ELISA; specific T cell populations were expanded using feeder cells and LCL and maintained in RPMI medium containing 10% FCS, 1% HuS, 25% MLA 144 supernatant and 20IU/ml rIL-2.

### HLA restriction analysis and definition of minimal epitopes

LCLs sharing one or more HLA class I alleles with the donor of interest were pre-loaded with peptide (1μg/ml, 1hour incubation followed by washing) and co-cultured overnight with specific T cell clones (20,000 LCL and 1–2,000 T cells/well). T cell recognition was measured by IFNγ ELISA. Minimal epitopes were predicted using the following algorithms: SYFPEITHI (http://www.syfpeithi.de), The Immune Epitope Database and Analysis Resource (IEDB; http://www.iedb.org) and Bimas (http://www-bimas.cit.nih.gov/molbio/hla_bind); T cell recognition of minimal epitope peptides was confirmed by IFNγ ELISA.

### Elispot assays

PBMCs were tested in ex vivo ELISPOT assays of IFNγ release against individual defined epitope peptides (final concentration 5μg/ml) as previously described [51]. An equivalent volume of DMSO and 10μg/ml PHA were added to separate wells as negative and positive controls respectively.

### Infection of primary B cells

To analyse events occurring shortly after EBV infection in vitro, primary B cells were isolated from donor PBMCs by positive selection using CD19 beads according to the manufacturer’s instructions (Dynal) and exposed to EBV (either supernatant from the B95.8 cell line, centrifuged and filtered to remove cellular debris, or purified recombinant wt2089 virus, quantified as previously described [46] and used at a MOI of 100:1) for 2hrs at 37°C, washed once and used as required.

### T cell recognition of newly infected B cells

Following EBV infection, B cells were co-cultured with specific T cell clones (20–40,000 B cells and 1–5,000 T cells/well) in 200μl RPMI containing 10% FCS and 40IU/ml IL-2, in 96U well plates. Uninfected B cells or B cells pre-pulsed for 1 hour with 1μg/ml of the relevant epitope peptide served as negative and positive controls respectively. Supernatant (~80μl) was harvested at time points between 24hrs and 14 days and stored frozen. An equivalent volume of fresh medium was replaced in the co-cultures. At the end of the time-course the supernatant from all time points was assayed concomitantly by IFNγ ELISA.

### EBV gene expression

RNA was prepared using a Nucleospin II kit (Macherey-Nagel) and subjected to an additional DNAse treatment using a DNAfree kit (Life Technologies); cDNA was then synthesised using QScript (VWR). All protocols were carried out according to the manufacturer’s instructions. Samples were then analysed using a high throughput 48:48 Dynamic Array IFC-Gene Expression system (Fluidigm). Absolute quantitation was achieved by generating a standard curve for each target gene using a dilution series of the AQ-plasmid standard which contained a single copy of each of 45 EBV and 3 cellular amplicons [34]. Immunoblotting was carried out as described previously [36] using mAbs to EBNA2 (PE2), LMP2 (14B7) and BHRF1 (5B11) and a polyclonal antibody specific for EBNA3B (Exalpha Biologicals).

### T cell inhibition of B cell outgrowth

Following EBV infection, B cells (20,000 cells/well) were seeded into 96U well plates in RPMI containing 10% FCS and 40IU/ml IL-2 and co-cultured with specific T cell clones at effector to target ratios between 2:1 and 0.031:1 (40,000 to 625 T cells/well) in triplicate wells. B cells cultured in the absence of T cells were included as a positive control for virus infection and transformation. Cultures were refed weekly by a half change of standard medium without cytokine supplement. Outgrowth was scored visually at ~4 weeks and the B cell identity of growing cultures confirmed by monoclonal antibody staining for CD19.

## Supporting Information

S1 Fig Identification of additional EBNA2- and BHRF1-specific CD8+ T cell responses. (A) Top left panel: In vitro expanded CD8-enriched polyclonal T cells from Donor 8 were screened against overlapping 20mer peptides spanning the complete unique sequence of EBNA2. Top right panel: Individual component peptides from pool 14 were screened for their ability to mediate IFNγ production by the CD8-enriched T cell population. Middle panel: HLA restriction analysis of the pool 14-specific response; LCLs sharing one or more class I alleles with Donor 8 (class I type in bold) were pre-loaded with peptides 14.2–14.4 (1μg/ml) and co-cultured overnight with a specific T cell clone. Results are expressed as the mean IFNγ concentration +/- SD for triplicate wells. Table: The three overlapping 20mer peptides (14.2–14.4; pooled) and the predicted minimal epitope were screened for their ability to mediate IFNγ production by the CD8-enriched T cell population. (B) Top left panel: In vitro expanded CD8-enriched polyclonal T cells from Donor 9 were screened for reactivity against overlapping peptides spanning BHRF1. Top right panel: Individual component peptides from pool 3 were screened for their ability to mediate IFNγ production by the total polyclonal T cell population. Middle panel: HLA restriction analysis of the pool 3-specific response. Table: Peptide 3.1 and the predicted minimal epitope were screened for their ability to mediate IFNγ production by the CD8-enriched T cell population. (C) and (D) Recognition of antigen endogenously expressed from recombinant vaccinia viruses (rVV). (C) LCLs of appropriate HLA class I type were infected with rVVs (modified vaccinia ankara, MVA) expressing EBNA2 or EBNA3B (control) and co-cultured overnight with TSS- (left panel) or QPR- (right panel) specific T cell clones. Results are expressed as the mean IFNγ concentration +/- SD for triplicate wells. (D) LCLs were infected overnight with rVVs expressing BHRF1 or TK- control, and then used as targets with ETF- (left panel) or SRV- (right panel) specific T cell clones in standard 5hr chromium release assays. Results are expressed as % specific lysis.
(PDF)

S2 Fig Analysis of EBNA1, EBNA3A- and EBNA3C-specific T cell recognition following EBV infection of B cells in vitro. (A) Left panels: Primary B cells (HLA-B*2705-, B35-positive) were infected with EBV (B95.8 supernatant) then co-cultured with latent antigen-specific (EBNA1: HPV/B35, EBNA3A: YPL/B35, EBNA3C: RRI/B*2705) T cell clones (20,000 B cells + 2000 T cells/well). Culture supernatant was harvested at the specified time points and the IFNγ concentration measured by ELISA; results are the mean of triplicate wells +/- SD. Right panels: T cell recognition of an established LCL from the same donor as the primary B cells -/+ cognate epitope peptide. (B) In parallel, primary B cells were infected with an EBNA2-KO virus then co-cultured with T cells and assayed as in (A).
(PDF)

S3 Fig BHRF1- and EBNA2-specific T cell recognition: peptide titrations. An HLA-A68, B*5501-positive LCL was pre-loaded with epitope peptide (top panel: ETF (BHRF1), bottom panel: RPT (EBNA2)) at concentrations between 10^−6^ and 10^-12^M, then co-cultured with specific T cell clones; recognition was assessed by IFNγ ELISA. *indicates recognition of LCL plus control peptide (RPT and ETF respectively) at 10^-6^M.
(PDF)

S1 Table Individual donor responses to EBNA2, EBNA-LP and BHRF1. (PDF)
