# Supplementary material for: Early T Cell Recognition of B Cells following Epstein-Barr Virus Infection: Identifying Potential Targets for Prophylactic Vaccination
Source: PLoS Pathog. 2016 Apr 20;12(4):e1005549. doi: 10.1371/journal.ppat.1005549 (PMC4838210; doi:10.1371/journal.ppat.1005549)
Supplement: S1 Fig — (A) Top left panel: In vitro expanded CD8-enriched polyclonal T cells from Donor 8 were screened against overlapping 20mer peptides spanning the complete unique sequence of EBNA2. Top right panel: Individual component peptides from pool 14 were screened for their ability to mediate IFNγ production by the CD8-enriched T cell population. Middle panel: HLA restriction analysis of the pool 14-specific response; LCLs sharing one or more class I alleles with Donor 8 (class I type in bold) were pre-loaded with peptides 14.2–14.4 (1μg/ml) and co-cultured overnight with a specific T cell clone. Results are expressed as the mean IFNγ concentration +/- SD for triplicate wells. Table: The three overlapping 20mer peptides (14.2–14.4; pooled) and the predicted minimal epitope were screened for their ability to mediate IFNγ production by the CD8-enriched T cell population. (B) Top left panel: In vitro expanded CD8-enriched polyclonal T cells from Donor 9 were screened for reactivity against overlapping peptides spanning BHRF1. Top right panel: Individual component peptides from pool 3 were screened for their ability to mediate IFNγ production by the total polyclonal T cell population. Middle panel: HLA restriction analysis of the pool 3-specific response. Table: Peptide 3.1 and the predicted minimal epitope were screened for their ability to mediate IFNγ production by the CD8-enriched T cell population. (C) and (D) Recognition of antigen endogenously expressed from recombinant vaccinia viruses (rVV). (C) LCLs of appropriate HLA class I type were infected with rVVs (modified vaccinia ankara, MVA) expressing EBNA2 or EBNA3B (control) and co-cultured overnight with TSS- (left panel) or QPR- (right panel) specific T cell clones. Results are expressed as the mean IFNγ concentration +/- SD for triplicate wells. (D) LCLs were infected overnight with rVVs expressing BHRF1 or TK- control, and then used as targets with ETF- (left panel) or SRV- (right panel) specific T cell clones in standa [file ppat.1005549.s001.pdf]

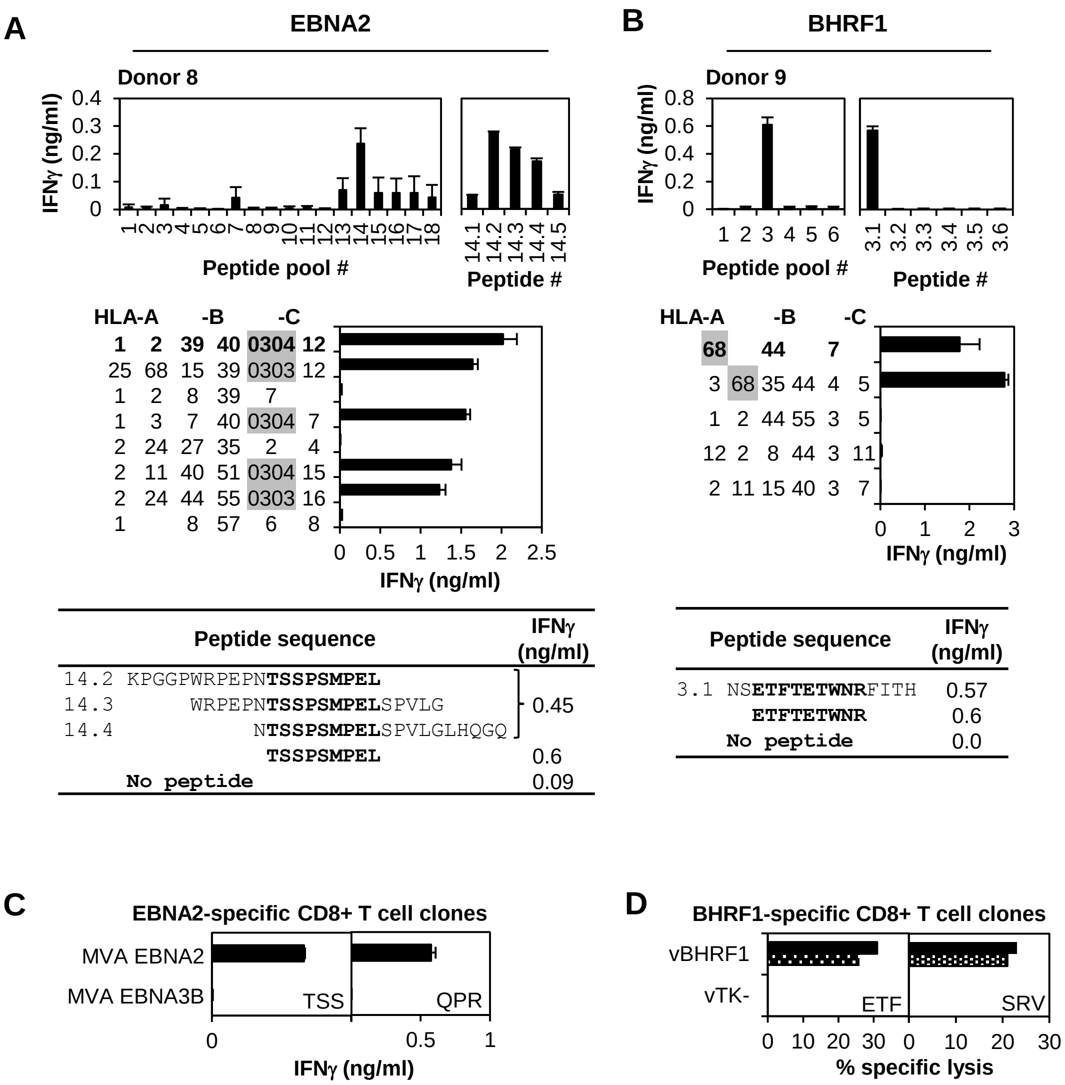
A
EBNA2
Donor 8
IFNγ (ng/ml)
0.4
0.3
0.2
0.1
0
1 2 3 4 5 6 7 8 9 10 11 12 13 14 15 16 17 18
Peptide pool #
14.1 14.2 14.3 14.4 14.5
Peptide #
HLA-A -B -C
1 2 39 40 0304 12
25 68 15 39 0303 12
1 2 8 39 7
1 3 7 40 0304 7
2 24 27 35 2 4
2 11 40 51 0304 15
2 24 44 55 0303 16
1 8 57 6 8
0 0.5 1 1.5 2 2.5
IFNγ (ng/ml)
Peptide sequence
IFNγ (ng/ml)
14.2 KPGGPWRPEPNTSSPSMPEL
14.3 WRPEPNTSSPSMPELSPVLG
14.4 NTSSPSMPELSPVLGLHQGQ
0.45
TSSPSMPEL 0.6
No peptide 0.09
B
BHRF1
Donor 9
IFNγ (ng/ml)
0.8
0.6
0.4
0.2
0
1 2 3 4 5 6
Peptide pool #
3.1 3.2 3.3 3.4 3.5 3.6
Peptide #
HLA-A -B -C
68 44 7
3 68 35 44 4 5
1 2 44 55 3 5
12 2 8 44 3 11
2 11 15 40 3 7
0 1 2 3
IFNγ (ng/ml)
Peptide sequence
IFNγ (ng/ml)
3.1 NSETFTETWNRFITH 0.57
ETFTETWNR 0.6
No peptide 0.0
C
EBNA2-specific CD8+ T cell clones
MVA EBNA2
MVA EBNA3B
TSS
QPR
0 0.5 1
IFNγ (ng/ml)
D
BHRF1-specific CD8+ T cell clones
vBHRF1
vTK-
ETF
SRV
0 10 20 30 0 10 20 30
% specific lysis
