# Supplementary figures and images for: Early T Cell Recognition of B Cells following Epstein-Barr Virus Infection: Identifying Potential Targets for Prophylactic Vaccination
Source: PLoS Pathog. 2016 Apr 20;12(4):e1005549. doi: 10.1371/journal.ppat.1005549 (PMC4838210; doi:10.1371/journal.ppat.1005549)

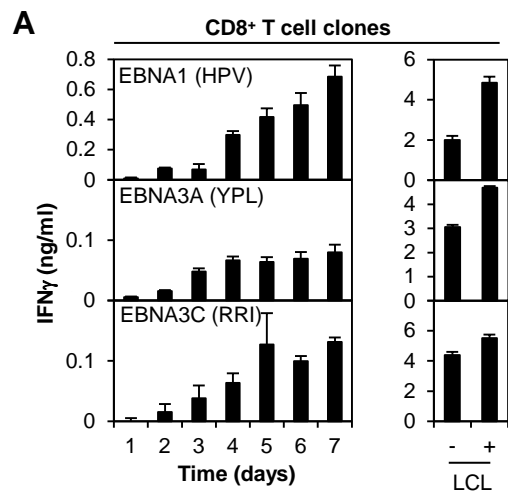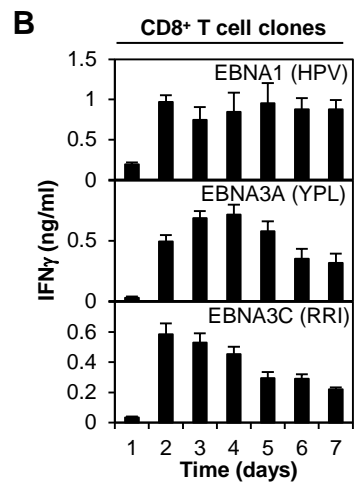

Supplement: S2 Fig — (A) Left panels: Primary B cells (HLA-B*2705-, B35-positive) were infected with EBV (B95.8 supernatant) then co-cultured with latent antigen-specific (EBNA1: HPV/B35, EBNA3A: YPL/B35, EBNA3C: RRI/B*2705) T cell clones (20,000 B cells + 2000 T cells/well). Culture supernatant was harvested at the specified time points and the IFNγ concentration measured by ELISA; results are the mean of triplicate wells +/- SD. Right panels: T cell recognition of an established LCL from the same donor as the primary B cells -/+ cognate epitope peptide. (B) In parallel, primary B cells were infected with an EBNA2-KO virus then co-cultured with T cells and assayed as in (A). (PDF) [file ppat.1005549.s002.pdf]

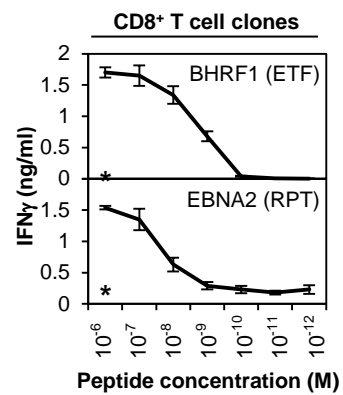

Supplement: S3 Fig — An HLA-A68, B*5501-positive LCL was pre-loaded with epitope peptide (top panel: ETF (BHRF1), bottom panel: RPT (EBNA2)) at concentrations between 10−6 and 10-12M, then co-cultured with specific T cell clones; recognition was assessed by IFNγ ELISA. *indicates recognition of LCL plus control peptide (RPT and ETF respectively) at 10-6M. (PDF) [file ppat.1005549.s003.pdf]
