# Supplementary material for: Early T Cell Recognition of B Cells following Epstein-Barr Virus Infection: Identifying Potential Targets for Prophylactic Vaccination
Source: PLoS Pathog. 2016 Apr 20;12(4):e1005549. doi: 10.1371/journal.ppat.1005549 (PMC4838210; doi:10.1371/journal.ppat.1005549)
Supplement: S1 Table — (PDF) [file ppat.1005549.s004.pdf]

S1 Table.

Individual donor responses to EBNA2, EBNA-LP and BHRF1

Donor		CD8			CD4		
**#**	**HLA type**	**EBNA2**	**EBNA-LP**	**BHRF1**	**EBNA2**	**EBNA-LP**	**BHRF1**
**1**	A2, A3, B7, C7<br>DR15, 51, DQ6	*SPL*⊥	-	nt	4,15,16*	QEP	nt
**2**	A2, 26, B38, 51, C12, 15<br>DR4, 53, DQ8	YHL	nt	nt	TPL	nt	nt
**3**	A2, 24, B44, 55, C0303, 16<br>DR7, 14, 52b, 53, DQ2, 5	RPT	-	-	FVG,PRS,11,17	QEP	NSE, PYY
**4**	A1, B7, 8,<br>DR15, 17, 51, 52a, DQ2, 6	-	-	-	PRS,PAQ	-	-
**5**	A26, B44, 58, C3, C7<br>DR14, 16, 51, 52b, DQ5	LAS**	-	-	-	QEP	-
**6**	A2, B55, 58, C0303, 7<br>DR4, 16, 51, 53, DQ5, 8	LAS	-	-	3,8,PRS	-	-
**7**	A1, 2, B44, 55, C0303, 5<br>DR4, 14, 52b, 53, DQ5, 7	RPT	nt	-	PRS	nt	PYY
**8**	A1, 2, B39, 40, C0304, 12<br>DR1, 13, 52b, DQ5, 6	TSS	-	SRV	FVG,PRS,11	-	-
**9**	A68, B44, C7<br>DR7, 11, 52b, 53, DQ2, 7	-	-	ETF	3,10,13,14	2	-
**10**	A3, 68, B7, 35, C4, 7<br>DR1, 15, 51 DQ5, 6	-	-	ETF	-	-	-
**11**	A1, 2, B57, 6<br>DR9, 13, 52b, 53, DQ2, 7	LAS	-	-	FVG,PRS,11	-	-
**12**	A1, B8, 57<br>DR7, 17, 52a, DQ2, 9	-	-	-	3,PRS,PAQ	2	-
**13**	A3, 31, B7, 38, C7, 12<br>DR7, 13, 52a, 53, DQ2, 6	YHL	-	-	PRS	2	-
**14**	A2, 29, B8, 40<br>DR4, 17, 52a, 53, DQ2, 8	-	-	-	VCR	-	-
**15**	A2, 24, B39, C6, 7<br>DR8, 15, 51, DQ4, 6	-	-	-	8	-	-
**16**	nt	*DVG*⊥⊥	-	-	FVG,PRS,PAQ	-	3
**17**	A1, 11, B7, 35, C4, 7<br>DR1, 15, 51, DQ5, 6	QPR	-	-	10	-	-
**18**	A2, 24, B27, 35, C2, 4<br>DR4, 53, DQ8	-	-	-	15,16	-	-
**19**	A2, 68, B35, 49, C4, 7<br>DR1, 12, 52b, DQ5, 7	*SMP*	-	ETF	PRS,11	2	-
**20**	A2, A0203#	nt	SLR	-	nt	-	-

Epitopes are identified by the first three amino acids of their sequence.
Italicised CD8 epitopes – minimal sequence not defined.
⊥No response to QPR epitope contained within 20mer.
nt not tested
*Numbers denote peptide pools recognised. Individual peptides within pools not screened.
** Response detected by Elispot only.
⊥⊥No response to LAS epitope contained within 20mer.
mini-typed for HLA-A2 only
